# Loci associated with spontaneous abortion in primiparous Holstein cattle

**DOI:** 10.3389/fvets.2025.1599401

**Published:** 2025-05-30

**Authors:** Emaly M. Suarez, Victoria C. Kelson, Jennifer N. Kiser, Kimberly M. Davenport, Brenda M. Murdoch, Allison L. Herrick, Holly L. Neibergs

**Affiliations:** ^1^Department of Animal Sciences, Washington State University, Pullman, WA, United States; ^2^Washington Animal Disease Diagnostic Laboratory, Washington State University, Pullman, WA, United States; ^3^Department of Animal, Veterinary and Food Sciences, University of Idaho, Moscow, ID, United States

**Keywords:** artificial insemination, cattle, dairy, embryo transfer, genomic selection, loci, spontaneous abortion

## Abstract

**Introduction:**

Spontaneous abortion (SA) in cattle refers to pregnancy loss occurring between days 42 and 260 of gestation. SA is costly, inefficient, and often leads to premature culling of cows.

**Methods:**

This study aimed to identify loci associated with SA in primiparous Holstein cows by conducting a genome-wide association analysis of cows bred via artificial insemination (AI; 679 controls and 69 cases that aborted) or that were embryo transfer (ET) recipients (236 controls and 41 cases) from a single dairy.

**Results:**

In AI-bred cows, 86 loci (413 single nucleotide polymorphisms or SNPs) and 168 positional candidate genes were associated (FDR <0.05) with SA, while in ET recipients, 4 loci (10 SNPs) and 16 positional candidate genes were associated (FDR <0.05) with SA. No SA-associated loci were shared between AI-bred or ET recipient cows, but nine associated loci (FDR <0.05) in AI-bred cows were shared with AI-bred heifers.

**Discussion:**

The difference in loci associated with AI-bred and ET recipient cattle may be due to differences in mechanisms associated with the maintenance of pregnancy between *in vivo* and *in vitro* derived embryos, or a larger sample size may be needed to identify additional shared loci. Identifying loci associated with SA in AI-bred and ET recipient cows provides an opportunity to enhance selection for reproductive efficiency in Holstein cattle.

## 1 Introduction

Spontaneous abortion (SA) or fetal loss typically occurs in 4 to 24% of pregnancies in primiparous cows (1–3). Spontaneous abortion results in increased culling of animals and reduced milk production, resulting in significant economic losses for the dairy industry. Keshavarzi et al. (4) found that SA reduced milk yield by 19.4% in cows that initiated lactation due to SA and by 7.3% in cows that experienced SA during lactation. Animals experiencing SA have an increased risk of retaining fetal membranes and other health issues, which may further reduce reproductive performance and increase costs to the industry (5–7).

Genomic selection for conception rate, daughter pregnancy rate, calving ability/ease (the ability of calves to be born unassisted), and calving interval (the time between the birth of two calves for the same cow) has been utilized to improve reproductive efficiency (8–10). In Holstein cattle, the conception rate improved by approximately 5% in heifers and 18% in cows between 2010 and 2022 (11, 12). Although the use of fertility traits in genomic selection has accelerated genetic progress for fertility, especially for embryonic loss, there is limited information regarding SA. Specifically, the genetic impact on SA risk in cows bred by artificial insemination (AI) compared to those that are embryo transfer (ET) recipients is not well understood. Recent reports have indicated the genetic effects of heifer genomics on SA for those bred by AI and those that were ET recipients, but there is limited research on SA in primiparous cows (13).

Some loci associated with fertility traits may differ between heifers and cows due to the distinct biological demands experienced by the two parities. For example, there are differences in the nutritional demands of a growing heifer compared to those of a lactating primiparous cow. The energy demand for heifers is focused on growth and puberty, while primiparous cattle must recover from parturition, lactate, and return to estrus. Milk production typically peaks between 60 and 100 days post-calving, coinciding with the time when cows are being bred (14). This creates a significantly greater nutritional demand on primiparous cows as they support their energy needs, the energy required for milk production, and, after conception, the energy demands of a developing fetus. These differences necessitate evaluating primiparous cows separately from heifers. To better understand how the use of ET affects SA compared to AI-bred cattle, the loci and positional candidate genes associated with SA in primiparous cows must be identified. Additionally, it is important to determine whether selecting for reduced fetal loss in AI-bred cattle can also improve calving rates in ET recipients by further evaluating these loci. Therefore, the objective of this study was to identify loci and genes associated with SA in US Holstein primiparous cows bred by AI or serving as ET recipients using a genome-wide association analysis (GWAA). These results will pinpoint loci that could be utilized in genomic selection indices and provide identification of specific genes associated with SA in cattle bred by AI or those that are ET recipients, leading to a better understanding of the fetal loss process.

## 2 Materials and methods

### 2.1 Study population and phenotypes

Health and breeding records for 5,750 primiparous Holstein cows were obtained using Dairy Comp 305 (Valley Agricultural Software, Tulare, CA, USA) records from a single dairy in southern Idaho. Data were recorded daily for breeding, production, and disease. The dairy consists of a milking herd of 2,300 cows housed in a dry lot with shade and fed a total mixed ration. Only cows that were pregnant to the first AI (*n* = 748) or were confirmed pregnant to the first ET (*n* = 277) were included in the study to eliminate possible confounding of phenotypes for embryonic loss. Cows were bred following a double ovulation synchronization protocol or after standing estrus prior to AI. There was no difference (*p* < 0.05, ANOVA) in the frequency of SA in cows bred by AI during observed estrus or following synchronization. Cows that were ET recipients received a double ovulation synchronization protocol with embryo transfer on day 7 post-estrus. Pregnancy was determined using trans-rectal ultrasound at 30 days post-breeding. Phenotypes were based on whether a cow maintained a pregnancy to ≥ 260 days of gestation (control) or spontaneously aborted between days 42 and 260 of gestation (case). Cases were identified by the cows returning to estrus following a confirmed pregnancy. AI controls were compared to AI cases, while ET controls were compared to ET cases. Four AI cows and three ET recipient cows were removed from the study as they experienced mastitis, metritis, metabolic issues, lameness, or respiratory disease between approximately day 30 before breeding and/or before calving. After filtering for these events, 679 AI controls, 69 AI cases, 236 ET controls and 41 ET cases remained in the study. All cows were at least 25 months of age at the beginning of their first lactation.

### 2.2 Genotyping and imputation

Genotypes from Zoetis (Kalamazoo, MI, USA) were imputed to a higher density utilizing Beagle (V. 4.1) and an in-house reference population of 4,800 US Holsteins genotyped with the Illumina (San Diego, CA, USA) BovineHD BeadChip (15). The 636,042 imputed single nucleotide polymorphisms (SNPs) had a call accuracy of >95% across the genome. Accuracy was determined by reducing the genotyping density of animals in the reference population to approximately 50,000 SNPs. Imputation was then performed on the lower density genotypes to restore them to the BovineHD BeadChip level for assessing the accuracy of imputation. Genotypes from the imputed SNPs were compared with the SNPs obtained from the initial genotyping using the BovineHD BeadChip.

### 2.3 Quality control

Prior to the GWAA, imputed genotypes were filtered for quality. SNPs were removed if they had a call rate <0.90, had a minor allele frequency (MAF) <0.01 or if they failed (*p* < 1 x 10^−100^) Hardy–Weinberg equilibrium testing. Primiparous cows bred by AI had 4,733 SNPs removed for call rate, 140,423 SNPs were removed for MAF, and 1,012 SNPs were removed for failing Hardy–Weinberg equilibrium testing. For primiparous cows receiving ET 4,658 SNPs were removed for call rate, and 151,385 SNPs were removed for low MAF. No SNPs failed Hardy–Weinberg equilibrium testing for cows that were ET recipients, nor were any cows removed for genotyping call rate (<0.90) or for differences between genotypic and phenotypic sex. After quality control was complete, a total of 1,025 cows remained for the analyses, with 489,927 SNPs remaining for cows bred by AI and 479,927 SNPs remaining for ET recipient cows.

### 2.4 Genome-wide association analysis

A GWAA was performed using the SNP and Variation Suite (SVS) software version 8.1 (70). An Efficient Mixed Model Associated eXpedited (EMMAX) statistical approach was used with an identity by state matrix (16). The general mixed model for EMMAX is expressed as γ = *Xβ*+*Zμ*+ε where y = the vector of observed phenotypic values, X = a matrix of fixed effects, β = regression coefficients, Z = a matrix containing the observed random effects, *u* = vector of random effects related to variants of allele substitutions in the population, and ε = residual effects (16). Associations with SA were identified when FDR <0.05 (17, 18). Dosage compensation for SNPs on the X chromosome were not accounted for in EMMAX.

Due to the unknown inheritance of SA in cattle, three inheritance models (additive, dominant, and recessive) were analyzed for AI and ET groups. The additive model identified associations where having two minor alleles (aa) was twice as likely to impact the phenotype (SA) compared to having no minor alleles (AA), and half as likely to have an impact when one minor allele (Aa) was present. The dominant model identified associations where having one or two minor alleles (Aa or aa) was likely to have more impact on the phenotype compared to not having a minor allele (AA). A recessive model identified associations where having two minor alleles (aa) was likely to have more impact on the phenotype compared to having one or no minor allele (Aa or AA).

A principal component analysis was performed to evaluate population stratification. Two distinct clusters within the population based on birth year were identified (Supplementary Figure S1). An ANOVA (*p* < 0.05) was used to test if birth year, service sire, and season of conception affected SA. The tested covariates with the associated *p*-values for each population are listed in Table 1. To ensure that the population stratification was accounted for, the genomic inflation factor lambda (λ_GC_) was calculated in SVS for each model. For AI-bred primiparous cows, no covariates were included as none of the tested covariates showed a significant (*p* < 0.05) effect on SA (Number of Sires used). In contrast, ET recipient primiparous cows had a difference (*p* < 0.05) in SA by season, therefore, this covariate was included in the analysis. Additional covariates, not identified by ANOVA as significant, were not included in the analysis to minimize false negative errors due to over-correction of possible population stratification.

**Table 1:** *P*-value of tested covariates for artificial insemination (AI) bred and embryo transfer (ET) recipient primiparous cows.

**Covariate**	**AI**	**ET**
Birth year	0.889	0.158
Service sire	0.841	0.141
Season of conception	0.238	2.10 × 10^−4^
AI Tech	0.802	NA
ET Tech	NA	0.127
Synched/standing	0.544	0.524

If multiple SNPs were associated with SA on a chromosome, loci were defined by a threshold of D'> 0.7 (19, 20). For each locus, positional candidate genes were identified within the average haplotype size of 30.5 kb, calculated using the method of Gabriel (21), 5′ or 3′ to the associated SNP based on the bovine ARS-UCD 1.2 genome assembly (22).

The proportion of variance explained by a SNP was calculated in SVS utilizing the notation of Further Optimization when Covariates are Present, as described by Segura (72) and Vilhjalmsson (73). The sum of the proportion of variance explained for all SNPs will exceed 100% due to SNPs within a locus not being independent. This calculation was used to evaluate the percentage of total variation observed in spontaneous abortion that can be attributed to these SNPs.

A measure of the strength of the association between having the risk allele and experiencing a SA, referred to as relative risk, was calculated utilizing the frequencies of the minor and major alleles (23). The following equations were utilized for calculating relative risk for SA: *a*(*a*+*b*)/*c*(*c*+*d*), where a is the minor allele frequency in cases, b is the minor allele frequency in controls, c is the major allele frequency in cases, and d is the major allele frequency in controls.

Heritability was estimated for SA using a genomic best linear unbiased predictor (GBLUP) analysis (24, 25) with the average information algorithm (AI-REML), which is a bivariate restricted maximum likelihood analysis (26, 71). The AI-REML GBLUP method calculates variance components that are then used to calculate heritability, as the pseudo-heritability estimated by EMMAX can over-inflate the estimate in limited sample sizes.

Literature and database searches were conducted to identify any shared genomic regions or genes associated with SA and other fertility and production traits. These traits included the fertility index in Nordic Red Cattle, which encompasses multiple traits such as the number of inseminations per conception, duration of the interval from calving to first insemination, days from first to last insemination, and 46-day non-return rate (27). Other fertility traits included cow conception rate (percentage of cows that are pregnant at each service), daughter pregnancy rate (percentage of cows that become pregnant during each 21-day period), days open, and early embryonic loss (20, 28–31). Additionally, a comparison was made using tissue expression data based on fertility status and expression in reproductive tissues (32, 33). Any shared loci or positional candidate genes will be listed and discussed in the Discussion section.

Additionally, the comparison of production traits was conducted in two ways: first, through a literature search for shared loci and positional candidate genes related to production traits such as milk yield and milk components like fat yield, fat percentage, protein yield, and protein percentage (34). Secondly, multiple GWAA were utilized on the population of animals in this study that had complete lactation records for the following three production traits: total milk, total fat, and total protein.

## 3 Results

The combined rate of SA for first-service Holstein primiparous cows in AI-bred and ET recipient groups was 10.7%. Cows bred by AI experienced an SA rate of 9.2%, while cows that were ET recipients had an SA rate of 14.8%. The SA rate of 9.2% for cows bred by AI is consistent with other reports where the rate of SA ranged from 2 to 12% (3, 6). The SA rate of 14.8% for ET recipient cows was slightly higher than previous reports of 10 to 13% SA associated with *in vitro*-produced embryos (35, 36).

### 3.1 Loci associated with spontaneous abortion in primiparous cows bred by artificial insemination

**Figure 1:**
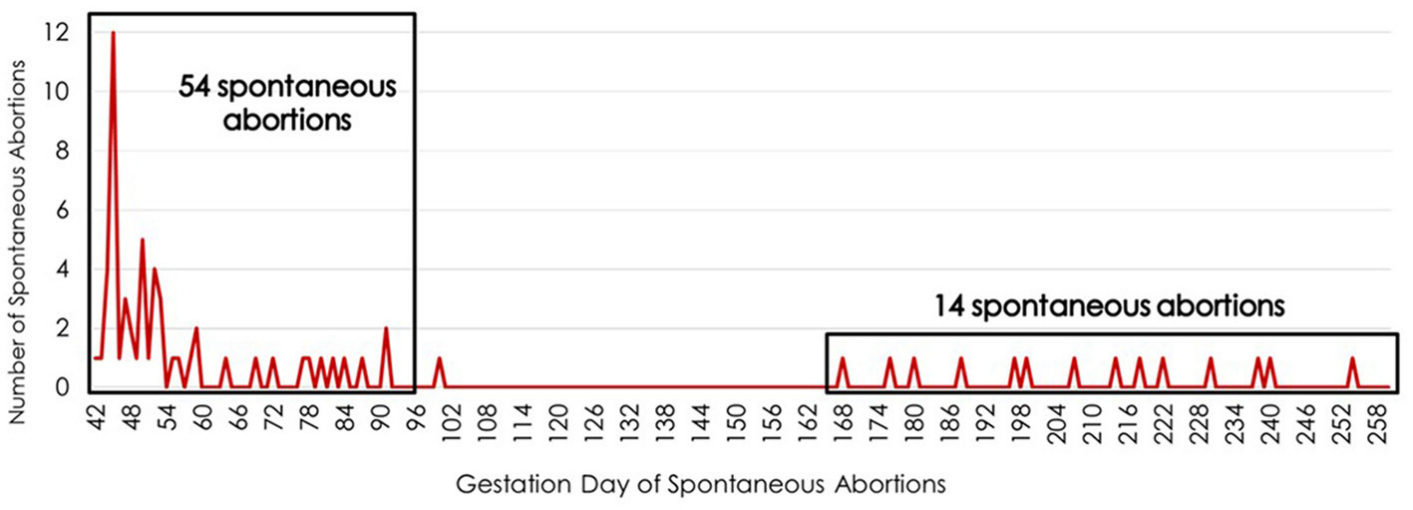
Timing of spontaneous abortions during the fetal period (days 42 to 260 of gestation) for primiparous cows bred by artificial insemination. The x-axis lists the gestation day while the y-axis indicates the number of spontaneous abortions occurring on each day of gestation during the fetal period.

The highest incidence of SA for primiparous cows bred by AI occurred between days 42 and 91 of gestation. The single highest day for SA was day 45 of gestation, with 12 cows aborting (Figure 1). These gestational days when SA was noted represent the day the cow returned to estrus after being identified as pregnant by trans-rectal ultrasound at approximately day 30 of gestation. The 54 SA occurrences between days 42 and 91 of gestation accounted for 78% of the fetal loss in this population. The second greatest interval of gestation associated with SA was between days 168 and 260, during which 20% of fetal loss occurred.

The λ_GC_ for SA in AI cows was 0.99 for the additive inheritance model, 1.01 for the dominant inheritance model, and 0.96 for the recessive model. The estimated heritability for SA in primiparous cows bred by AI was 0.03 (±0.053). As the standard error for the heritability estimate is larger than the estimate itself, this provides low confidence in the estimated heritability. Since the majority of the loci associated with SA were recessive, and heritability measures additive genetic variance, it is unsurprising that the heritability estimate for SA is low. This estimated heritability for SA is similar to that reported for primiparous cows at 0.09 (±0.08) in a previous study (3) and for other reproductive traits such as the number of services, days from calving to first service, days open, and calving interval, which have been reported to be 0.040 ± 0.017, 0.034 ± 0.011, 0.053 ± 0.019, and 0.056 ± 0.014, respectively (3, 37). Rahbar et al. (38) also reported similar heritability estimates for several fertility traits, such as days open (0.016) and gestation length (0.123). Two other traits with reported heritability estimates comparable to that calculated in this study include the interval from first calving to first insemination (0.013) and the interval from first to successful insemination for primiparous cows (0.038) (39).

**Figure 2:**
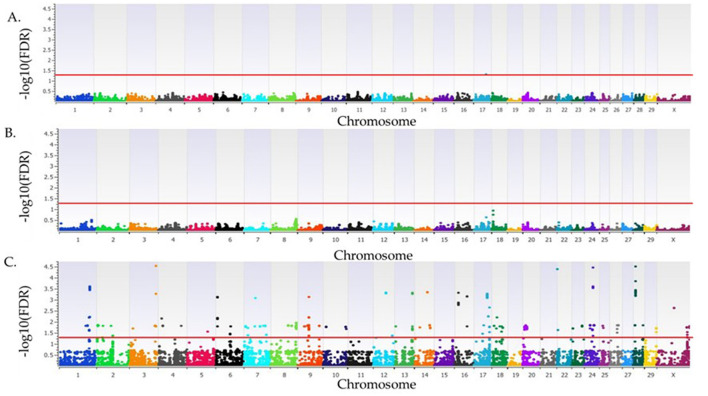
Loci associated with spontaneous abortion in Holstein primiparous cows that were bred by artificial insemination. The associations for the additive, dominant and recessive models of inheritance are represented in **(A–C)**, respectively. All plots have the Bos taurus chromosome on the x-axis, and the -log10FDR-value on the y-axis. The red line indicates the threshold for an association (FDR <0.05) with spontaneous abortion. The Y chromosome is absent as all animals were female.

In the recessive model, there were 85 loci associated (FDR <0.05) with SA (Figure 2, Supplementary Table S1). The most significant loci associated with SA in the recessive model were on BTA3 at 110 Mb (FDR = 2.9 × 10^−5^), BTA22 at 3 Mb (FDR = 4.7 × 10^−5^), BTA24 at 37 Mb (FDR = 3.5 × 10^−5^), and BTA28 at 9 Mb (FDR = 1 × 10^−4^) and 39 Mb (FDR = 3.07 × 10^−5^). Positional candidate genes at these loci include: *SFPQ, ZMYM4, KIAA0319L, RBMS3, DLGAP1, MTR, TRNAE-UUC, LOC112444209, LOC112446145, LOC618220*, and *LOC107131937*. An additional 156 positional candidate genes were identified as associated with SA, for a total of 168 positional candidate genes associated with SA in primiparous cows bred by AI. The positional candidate genes were grouped into 16 functional groups as shown in Supplementary Table S2.

The highest proportion of phenotypic variance explained for the primiparous cows bred by AI was 5.6% from the locus on BTA28 at 9 Mb, with two positional candidate genes, *MTR* and *TRNAE-UUC*. Additionally, 21 loci representing 45 positional candidate genes, listed in Table 2, had a proportion of variance explained >3%. The average relative risk for the most significant SNPs associated with SA in primiparous cows bred by AI was 2.20 (Supplementary Table S3). The four positional candidate genes of the most significant loci are further described below.

**Table 2:** Loci and positional candidate genes associated with spontaneous abortion in cows bred by artificial insemination with a proportion of variance explained >3%.

**BTA^a^**	**Locus**	**POV %^b^**	**Positional candidate gene(s) for locus^c^**
1	4	3.2	*-*
1	5	4.3	*-*
3	12	5.2	***ZMYM4**, KIAA0319L, SFPQ, LOC112446145, LOC618220*
4	13	3.1	***SLC25A13**, LOC112446491, LOC514680*
6	17	4.0	***SYNPO2, LOC101906469***
7	21	4.0	***TXNDC15, PITX1**, PCBD2, CATSPR3, LOC112447641*
9	32	4.0	***LOC528043***
12	37	3.9	*LOC112449132*
13	41	3.9	***SLC13A3, EYA2, ZMYND8**, TRNAR-CCU, TP53RK, LOC112449242, LOC101905203, LOC112449409, LOC104973934*
14	43	3.9	*-*
16	46	3.9	*-*
16	47	4.0	*-*
17	50	3.7	***NCOR2***
17	51	3.9	***CUX2, SH2B3, BRAP**, PHETA1, ATXN2, LOC513508*
17	52	3.2	*LOC100848596, LOC107133302*
16	55	3.4	*GRK3, LOC112442007*
18	58	3.2	*LOC785669*
22	65	5.3	*-*
24	71	4.3	***COLEC12, CLUL1**, CETN1, TYMS, LOC112444249*
24	73	5.3	*LOC786055*
28	81	5.6	***MTR**, TRNAE-UUC*
X	84	3.4	***SH3BGRL***

^a^Bos taurus chromosome. ^b^The proportion of variance (POV) explained for the 23 loci with a POV >3%. ^c^Positional candidate genes located within 30.5 kb on either side of the SNP(s) associated with each locus. Bolded gene names represent genes where the SNP is located within the gene.

Positional candidate genes associated with SA in primiparous cows bred by AI were differentially expressed in the endometrium and in different cell types of the bovine placenta (Supplementary Figure S2) during the embryonic and fetal periods. Twenty-eight positional candidate genes were differentially expressed in high fertility compared to sub-fertile heifers at day 17 of gestation (32). An additional 42 positional candidate genes were expressed in the reproductive tissues of cattle, based on single-cell data during the embryonic and fetal periods, which support their involvement in pregnancy (Table 3; Supplementary Figure S2) (33).

**Table 3:** Positional candidate genes for spontaneous abortion shared with fertility traits.

**References^a^**	**Trait**	**Positional candidate genes^b^**
Moraes et al. (32)	Differentially expressed genes based on fertility status in heifers	*ARHGAP28, ASXL3, ATRNL1, C3H1orf109, DAO, ELMO2, EYA2, FAM83B, GPRC5B, KIAA0319L, LAMA1, LOC101905203, LOC786974, MSX2, MTR, PAPD5, PDE4A, REPS2, RSPO1, SH3BGRL, SMAD6, SOBP, SOX5, SSBP2, SVOP, TLR4, TXNDC15, TYMS*
Davenport et al. (33)	Gene expression in cattle placenta	*AMMECR1L, AP1M1, ARHGEF2, ATXN2, B4GALT1, CD24, CDC37, CDCA8, COLEC12, CPQ, CTPS2, ELMO2, ENC1, FAM83B, GPRC5B, GRAMD1A, LAMA1, MAP3K2, MTR, NCOR2, NOL7, NSA2, PITX1, POLR2D, RANBP9, RBBP7, REPS2, RSPO1, SFPQ, SH2B3, SH3BGRL, SMAD6, SOBP, SOX5, SSBP2, SVOP, TPP2, TXNDC15, TYMS, VPS13B, ZMYM4, ZMYND8*
Galliou et al. (20)	Heifer embryonic loss	*ADCY7, AP1M1, COLEC12, CPQ, GFM2, LAMA1, METTL21C, NCOR2, OPRM1, SIRT5, SLC25A13, SVOP, TPP2, TYMS*
Kiser et al. (31)	Primiparous embryonic loss	*ADCY7, AP1M1, CDH13, CETN1, CLUL1, COLEC12, ELMO2, ERCC3, EYA2, GFM2, KIAA1671, LAMA1, NCOR2, NSA2, OPRM1, SLC13A3, TLR4, TPP2*

^a^Citation for the fertility study where positional candidate genes for spontaneous abortion were identified as associated, enriched for differentially expressed traits. ^b^List of positional candidate genes associated with spontaneous abortion identified in previous studies.

### 3.2 Association of spontaneous abortion in primiparous cows that were embryo transfer recipients

**Figure 3:**
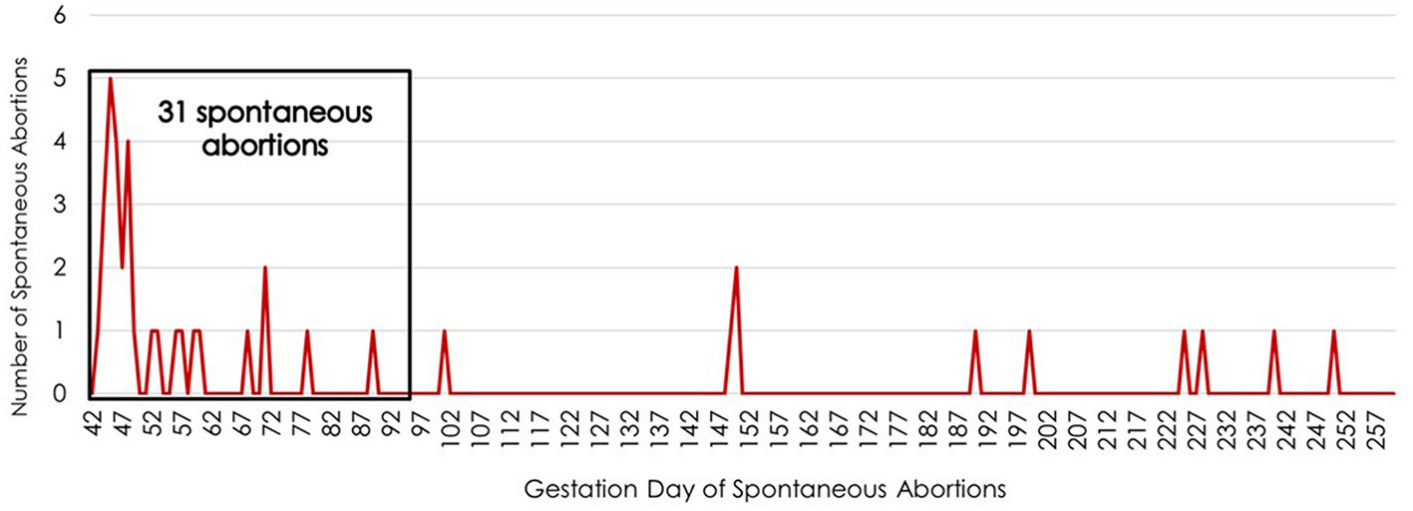
Timing of spontaneous abortions during the fetal period (days 42 to 260 of gestation) for primiparous cows that were embryo transfer recipients. The x-axis lists the gestation day while the y-axis indicates the number of spontaneous abortions occuring on each day of gestation during the fetal period.

Primiparous cows that were ET recipients also experienced the greatest number of SA on day 45 of gestation (Figure 3). Seventy-six percent of the SA in ET recipients occurred during the first trimester.

**Figure 4:**
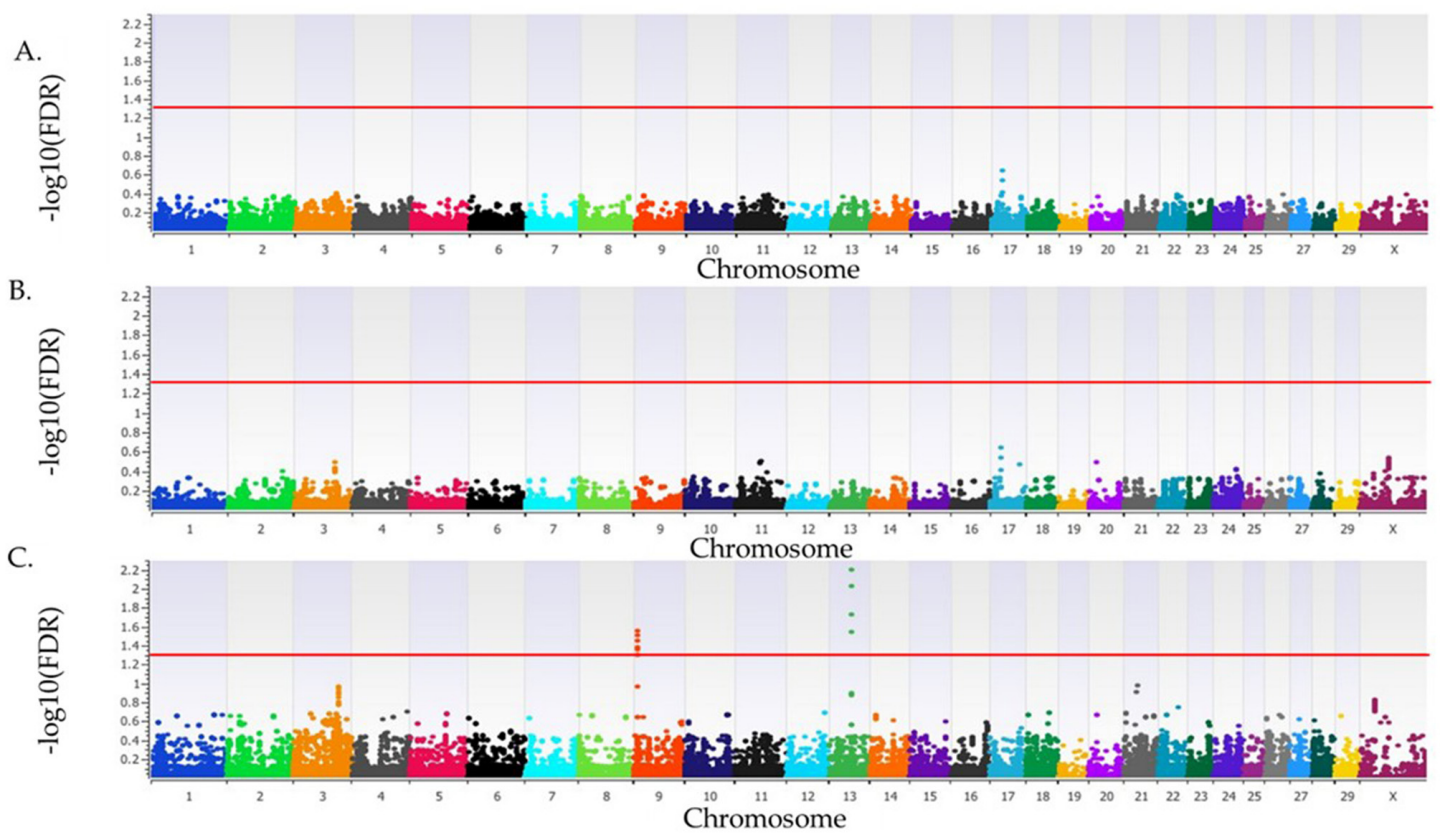
Loci associated with spontaneous abortion in Holstein primiparous cows that were embryo transfer recipients. The associations for the additive, dominant and recessive models of inheritance are represented in **(A–C)**, respectively. All plots have the Bos taurus chromosome on the x-axis, and the -log10FDR-value on the y-axis. The red line indicates the threshold for an association (FDR <0.05) with spontaneous abortion. The Y chromosome is absent as all animals were female.

The λ_GC_ for SA in cows that were ET recipients was 1.02, 1.02, and 1.03 for the additive, dominant, and recessive inheritance models, respectively. No loci were associated with SA in cows that were ET recipients in the additive or dominant inheritance models, but four loci were associated (FDR <0.05) with SA in the recessive inheritance model on BTA9 and BTA13 (Figure 4, Table 4). There were 14 positional candidate genes within the two loci on BTA13 (Table 4). All four loci associated with SA in ET recipient primiparous cows had a proportion of variance explained >3%. The smallest variance explained was 8.7%, belonging to the locus on BT 13 at 46 Mb, while the largest proportion of variance explained was 10.5% for the locus on BTA13 at 47 Mb. The positional candidate genes for these loci can be seen listed in Table 4.

**Table 4:** Loci associated with spontaneous abortions in cows that were embryo transfer recipients.

**BTA^a^**	**Position (Mb)^b^**	**FDR^c^**	**FA Freq^d^**	**POV^e^**	**Relative Risk^f^**	**Positional candidate gene(s) for locus^g^**
9	8	0.028	0.90	0.088	2.24	*-*
9	8.1	0.044	0.90	0.091	2.16	*-*
13	46	0.029	0.86	0.087	2.31	***WDR37, IDI1, GTPBP4, LOC11244939, LOC107133051, LOC101905075, LARP4B, LOC112440306, LOC112449304, LOC101905155**, LOC101904942*
13	47	0.006	0.76	0.105	2.17	***RASSF2, LOC112449419, SLC23A2**, PRND*

^a^Bos taurus chromosome. ^b^Location of SNPs measured by the numbered nucleotides in the ARS-UCD 1.2 reference genome assembly. ^c^FDR corrected P-value for each SNP associated with SA. ^d^Favorable allele (FA) frequency in the population. ^e^The proportion of variance (POV) explained for each SNP associated with spontaneous abortion. ^f^The calculated relative risk for the most significant SNP representing a locus. ^g^Positional candidate genes located within 30.5 kb on either side of the SNP(s) associated with each locus. Bolded gene names represent genes where the SNP is located within the gene.

Loci and positional candidate genes associated with SA were also compared with production traits to identify possible correlations to determine if selection for improved fertility would negatively impact selection for production traits. The loci and positional candidate genes associated with SA in AI bred and ET recipient primiparous cows were not associated (p <0.05) with dairy production traits. These traits included total milk, total fat, total protein, milk yield, fat yield, fat percentage, protein yield and protein percentage as mentioned in the materials and methods. This indicates that selection for production traits while selecting for reduced SA could be done simultaneously without reducing genetic progress in production traits.

## 4 Discussion

In cattle, ET has been found to be associated with abnormal morphology of the embryo, abnormal placentation, and elevated immune responses compared with pregnancies established by natural service or AI (40–43). Studies in mice have found that embryos resulting from ET have increased placental weight, a reduced fetal-to-placental weight ratio, and significantly larger tissues when compared to naturally conceived controls (42). ET also increases epigenetic perturbations that could potentially lead to adverse neonatal and long-term health outcomes in offspring (42). *In vitro*-produced embryos have a more fragile zona pellucida, reduced intracellular communication, a higher incidence of chromosome abnormalities, errors of imprinting, a slower growth rate, higher thermal sensitivity, lower inner cell mass/trophectoderm ratio, and differences in gene expression compared to *in vivo* embryos (43). The differences between *in vivo*-derived embryos and *in vitro*-produced embryos could be reflected in the different genes and loci associated with SA.

The identification of loci and positional candidate genes provides added information to pursue in understanding how these genomic regions are essential for the maintenance of pregnancy. Genomic mechanisms that influence pregnancy can result from modifications to positional candidate genes that alter gene function or expression, changes in regulatory element sites, or changes in methylation or chromatin accessibility that affect transcription.

### 4.1 Comparison of loci associated with spontaneous abortion in cows bred by artificial insemination and previous fertility studies

One locus on BTA17 at 51 Mb was associated (FDR = 0.049) with SA in the additive model with the positional candidate gene nuclear receptor corepressor 2 (*NCOR2)* (Figure 2, Supplementary Table S1). The *NCOR2* gene codes for a nuclear corepressor that interacts with multiple transcription factors. This corepressor orchestrates proliferation and differentiation during B cell development and is critical for fetal development (44). There were six loci associated with SA in cows bred by AI that were previously identified as associated with fertility traits. For a locus to be determined to be shared, the associated SNPs were required to be within a haplotype (31 kb) of each other. Two of these six loci, located on BTA1 and BTA4, were shared with genomic regions identified by Cole et al. (28) for daughter pregnancy rate (DPR) in Holstein cows. The sharing of loci between DPR and SA may be due to similar physiological processes necessary for the initiation and maintenance of pregnancy in cattle. A locus on BTA16 associated with cow conception rate to the first service and number of times bred in Holstein cattle was also associated with SA (31). A study by Höglund et al. (45) identified two loci (BTA2 at 46 Mb and BTA29 at 48 Mb) that were associated with fertility traits in Nordic cattle and shared with SA in this study. The locus on BTA2 was also shared with a genomic region identified for the Nordic female fertility index and days from first to last insemination in Nordic Holstein, Nordic Red, and Jersey cows (45). The Nordic female fertility index is a multi-trait index based on data collected on dairy cattle in Denmark, Sweden, and Finland and includes the number of inseminations required per pregnancy in cows and heifers, the length in days of the interval from calving to first insemination in cows, days from first to last insemination in cows and heifers, 56-day non-return rate in cows and heifers, and heat strength in cows and heifers (45). A locus on BTA12 at 52 Mb was also associated with a fertility index in Nordic Red cattle and with SA in Holstein cattle (27). Finally, a locus on BTA29 that was associated with SA was also associated with the number of inseminations per conception and days from first to last insemination in cows and heifers in Nordic Holstein, Nordic Red, and Jersey cows (45). Additionally, there were nine loci (BTA1 at 128 Mb, BTA5 at 85 Mb, BTA7 at 6 Mb, BTA7 at 85 Mb, two on BTA13 at 75 Mb, BTA17 at 35 Mb, BTA18 at 9 Mb, and BTA23 at 43 Mb) associated with SA in heifers bred by AI (13) and primiparous cows bred by AI. This indicates that shared genomic regions across parities could be used for genomic selection to help lower the risk and occurrences of SA.

There were 168 positional candidate genes, representing 86 loci, associated with SA in cows bred by AI, of which 68 were previously identified as having a role in fertility (Table 3). Two positional candidate genes (*FST5* on BTA17 and *CDH13* on BTA18) have been identified as positional candidate genes associated with SA in a separate population of Holstein cattle and were associated with embryonic loss (3, 20). The cadherin 13 (*CDH13*) gene, identified as a positional candidate gene for SA in AI bred cows, encodes a calcium-dependent cell adhesion, cadherin protein that protects vascular endothelial cells from apoptosis due to oxidative stress (46, 47). *CDH13* plays a role in cell migration and acts as a transcription repressor as a tumor suppressor gene (48–50). *CDH13* is correlated with trophoblastic invasion anomalies that lead to fetal growth restriction in humans, which can cause fetal demise (51, 52). Other studies have found that *CDH13* plays a role in early reproductive development and cholesterol biosynthesis (46). There were 23 positional candidate genes for SA in AI bred cattle that have previously been identified as enriched for embryonic loss and one (*EYA2*) that was enriched for SA in Holstein cattle (3, 20, 31). An additional 14 genes (*SOX5, LOC101904842, LOC100847115, EYA2, LOC101905203, ZMYND8, LOC104973934, LOC112449409, CDH13, PHACTR1, AP1M1, LOC112447625, FAM32A*, and *TRNAE-UUC)* were reported as positional candidate genes associated with SA in Holstein heifers bred by AI (13).

The four positional candidate genes of the most significant loci include the splicing factor proline and glutamine-rich (*SFPQ*) gene, which encodes an RNA-binding protein that is critical for maintaining transcriptional elongation of long genes and enabling histone deacetylase binding activity. Histone deacetylases are enzymes that catalyze the removal of acetyl functional groups from lysine residues of both nonhistone and histone proteins (53). The removal of epigenetic modifications regulates chromatin structure and transcription, while the deacetylation of non-histones controls a variety of cellular processes (53).

The second positional candidate gene from the most significant loci for SA is the RNA binding motif single-stranded interacting protein 3 (*RBMS3*) gene, which is involved in DNA replication, gene transcription, cell cycle progression, and apoptosis (54). This gene influences the regulation of microRNA expression or stabilization, inhibits the Wnt/β-catenin pathway, and affects other epithelial–mesenchymal transition-related transcription factors (55).

The third positional candidate gene is DLG associated protein 1 (*DLGAP1*), which is a part of the DLGAP family of proteins that act as scaffold proteins in the brain (56). These proteins play a vital role in synaptic scaling by regulating the turnover of both metabotropic and ionotropic glutamate receptors in response to synaptic activity (56). *DLGAP1* is specifically involved in megakaryocyte biology and platelet function, potentially giving a proliferative advantage in hematopoietic cells (57).

Lastly, the fourth positional candidate gene is LOC107131937, a pseudogene of the small ribosomal subunit protein uS12 that is conserved across all domains of life (58). The ribosomal protein S12 is a component of the decoding center of the 30S ribosomal subunit and is involved in transfer RNA selection (59). These transfer RNAs are key in synthesizing proteins, serving as an adapter or link between the messenger RNA (mRNA) molecule and the growing chain of amino acids that make up the protein (60). Pseudogenes can be either functional or non-functional; the status of this pseudogene is currently unknown. If it is functional, it could impact the synthesis of important proteins that affect SA. Holstein infertility haplotypes have also been discovered, which are significant due to their association with infertility, embryonic loss, and stillbirth in Holstein cattle. This makes it important to determine if any SA-associated loci found in this study may also correspond to several identified Holstein infertility (HH) haplotypes. Several Holstein infertility haplotypes (HH) have been identified and are currently being tested to reduce infertility. These haplotypes include HH1 to HH6 (61) and are located on BTA5 at 63 Mb (HH1), on BTA1 at 94 Mb (HH2), on BTA8 at 95 Mb (HH3), on BTA1 at 1.3 Mb (HH4), on BTA9 at 92 Mb (HH5), and on BTA16 at 29 Mb (HH6) (62, 63). The nearest locus associated with SA in primiparous cows bred by AI was approximately 1.2 Mb away from HH5 on BTA 9. The next closest loci were four loci on BTA 8 that ranged from 8 Mb to 22 Mb away from HH3. Additionally, there was one locus that was 19 Mb away from HH2 on BTA 1, one locus that was 13 Mb away from HH6 on BTA16, and another that was 22 Mb away from HH1 on BTA5. Any other loci associated with SA were over 22 Mb away from these identified Holstein haplotypes, indicating independent regions.

### 4.2 Comparison of loci associated with spontaneous abortion in cows bred as embryo transfer recipients and associated or enriched with other fertility traits

Sixteen positional candidate genes were associated with SA in cows that were ET recipients, with five (*SLC23A2, IDI1, PRNP, RASSF2*, and *GTPB4*) previously identified as having roles in fertility. Solute carrier family 23 member 2 (*SLC23A2*) encodes a sodium-dependent vitamin C transporter linked to spontaneous preterm birth in women, with research suggesting that genetic variants may increase the risk of preterm delivery (64). *IDI1* was previously reported as a leading-edge gene enriched with embryonic loss in Holstein heifers (20). Ras association domain family member 2 (*RASSF2*) is a tumor suppressor gene that regulates Ras signaling (65). The GTP binding protein 4 (*GTPBP4*) encodes a small GTP-binding protein located in the nucleus and is involved in signal transduction (66). There were no loci identified as associated with SA in primiparous cows receiving ET that were on the same chromosome as any of the six Holstein infertility haplotypes mentioned earlier.

### 4.3 Comparison of loci associated with spontaneous abortion in cows that were embryo transfer recipients and are differentially expressed

Three genes (*RASSF2, GTPBP4*, and *IDI1*) have been previously reported to be differentially expressed in the endometrium of pregnant beef heifers compared to non-pregnant, high-fertility beef heifers (32). *IDI1, PRNP*, and *LARP4B* were expressed in the reproductive tissues of cattle with pregnancies generated via embryo transfer, thus supporting their role in the function of a normal pregnancy (33).

Research is ongoing to determine the genomic differences between SA in AI-bred and ET-recipient cattle. There was no overlap of loci or genes associated with SA in cows bred by AI with those associated with SA in ET-recipient cows. This lack of overlap could be due to insufficient power (power = 0.2) to detect an association or due to distinct physiological, biological, and genomic regions essential for pregnancy establishment and maintenance in AI-bred and ET recipients. Lower statistical power also increases the risk of false negatives in the analysis; therefore, further studies need to be conducted with larger sample sizes to confirm these results. To the author's knowledge, there are no other association studies that have investigated loci associated with fetal loss in ET-recipient cattle, but there have been studies investigating embryonic loss in ET recipients. Similar results have been identified in loci associated with embryonic loss in cows and heifers bred by AI and those that were ET recipients (67) and are consistent with physiological studies that have identified differences between AI-bred and ET-recipient pregnancies.

For primiparous cows bred by AI and those receiving ET, the greatest number of SA (as indicated by cows returning to estrus) occurred on day 45 of gestation. This could indicate possible issues or deformities of the placenta that lead to SA. Functional insufficiency of the placenta often results in increased offspring mortality (68). The gestational interval between days 28 and 60 is a pivotal period for the development of the placenta (69). This period of gestation requires substantial growth and differentiation of embryonic and trophoblastic cells, with significant communication between the dam and the embryo occurring (69). After day 30 of gestation, a firm connection between fetal cotyledons and maternal caruncles occurs, with the fetus developing a distinguishable adult organ system by day 60. Fetal loss during this period can result from developmental deficiencies such as inadequate placentation, difficulty transitioning from amniotic to allantoic nutrition, changes in placental vascularization, and underdevelopment of the embryo or fetus (69). Twenty-five percent of pregnancies from *in vitro*-produced embryos are reported to be lost between days 28 and 60 of gestation (69). In these embryos, there is a clear underlying physiology of placental abnormalities that contribute to the high incidence of pregnancy loss during this period.

Other factors can impact SA, such as infectious diseases, nutritional status, and overall management. In this study, we accounted for infectious diseases and some nutritional issues such as ketosis by excluding animals from the study that were recorded as experiencing these events during their pregnancy. However, not everything can be carefully managed or recorded in the dairy's records (such as body condition score), so there is potential for confounding variables to go undetected and potentially impact the occurrence of SA.

A future direction for the analysis of SA with a greater number of cases would be to split the gestational window into two categories to include early losses (approximately 42 to 120 days) and late losses (121–260 days). As reported in the results, there was a greater number of SA that occurred between days 42 and 120 for both primiparous cows bred by AI and those that were ET recipients. Further analysis focusing on SA occurring between days 42 and 120 could also be beneficial, as early losses are more often linked to genetic factors than late-term losses. A future study examining these early and late losses could be very helpful in identifying genetic regions and biological components that impact SA losses at different times during gestation.

## 5 Conclusions

This study identified loci and positional candidate genes associated with SA in Holstein primiparous cows bred by AI and those that were ET recipients. While physiological differences between AI and ET services have previously been identified, this study further supports the existence of physiological differences in the maintenance of pregnancies from embryos produced by cows bred by AI compared to those that received ET, as evidenced by the different genomic regions identified in the GWAA. None of the loci identified for cows bred by AI and those receiving ET overlapped with reported loci associated with dairy production traits. This suggests that SA and dairy production traits can be selected for simultaneously without a negative correlated response. By identifying the loci and positional candidate genes associated with SA in AI bred and ET recipient cows, this study enhances the understanding of the underlying factors that contribute to SA in cattle.

There were six loci associated with SA in AI bred cows and five loci associated with SA in ET recipients that have previously been identified as associated with either SA or embryonic loss in cattle. These shared regions with embryonic loss could be due to the similarity of pregnancy loss in the SAs occurring at day 45 of gestation for both primiparous cows bred by AI and those that were ET recipients. Seven loci were shared with fertility traits in previous GWAA studies, and 18 positional candidate genes were related to pregnancy during the fetal period. Sixty-seven positional candidate genes for SA were differentially expressed in reproductive tissues during the fetal stage of pregnancy, supporting their role in pregnancy initiation or maintenance.

The occurrence of spontaneous abortion in Holstein dairy cattle results in higher culling rates, longer calving intervals, and decreased milk production. This makes SA a substantial economic cost to producers each year. By utilizing identified genomic regions associated with SA in AI bred and ET recipients, this study provides a basis for improving reproductive efficiency through genomic selection.

## Data Availability

The datasets presented in this study can be found in online repositories. The names of the repository/repositories and accession number(s) can be found below: https://osf.io/qym4e/, 10.17605/OSF.IO/QYM4E.
