# Supplementary material for: Loci associated with spontaneous abortion in primiparous Holstein cattle
Source: Front Vet Sci. 2025 May 30;12:1599401. doi: 10.3389/fvets.2025.1599401 (PMC12163616; doi:10.3389/fvets.2025.1599401)
Supplement: Supplementary file 1 [file Table_1.docx]

Supplementary Material


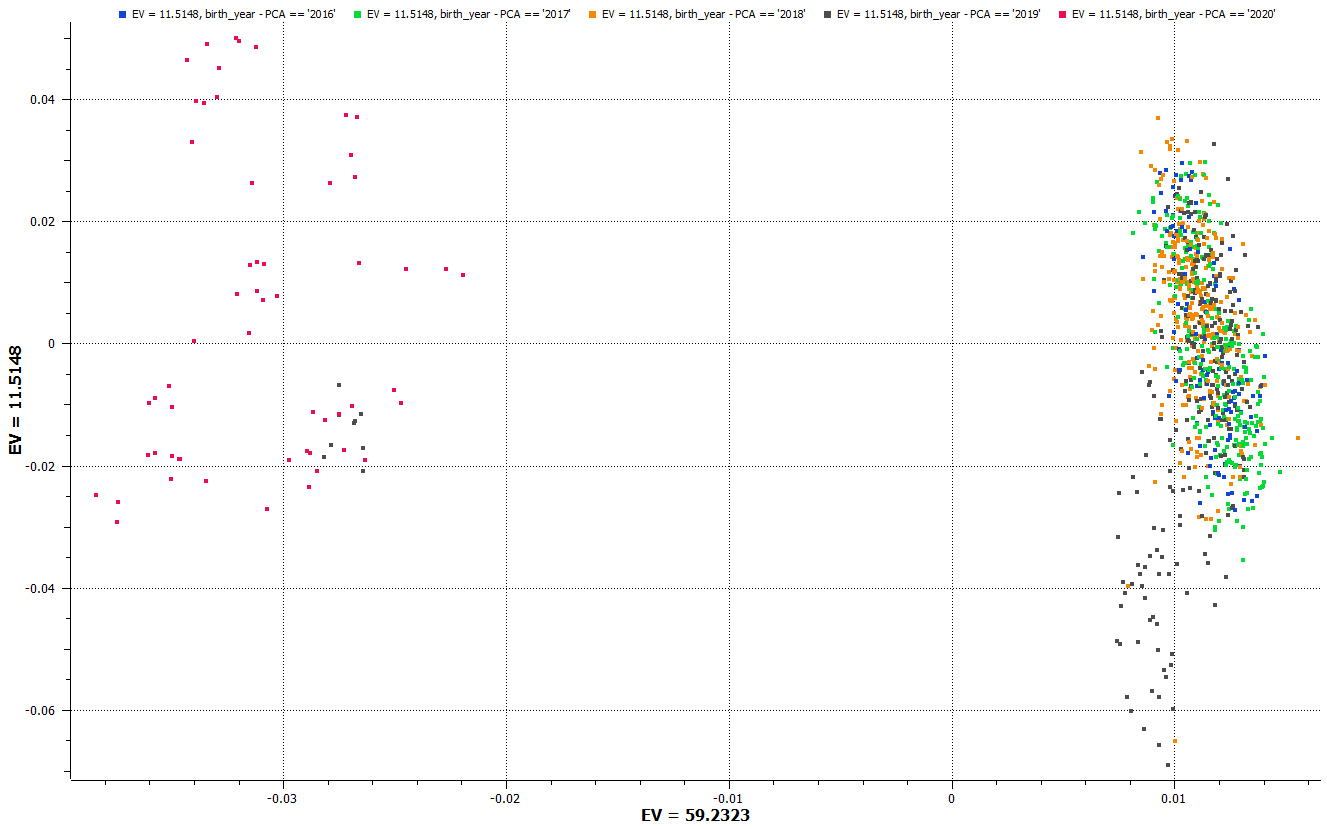


**Figure S1**: Principal component analysis to assess population stratification of primiparous cows, with different colors representing different years of birth colored by birth year. Royal blue represents cows born in 2016, green represents cows born in 2017, orange represents cows born in 2018, black represents cows born in 2019 and red represents cows born in 2020.

**Table S1**: Loci associated with spontaneous abortion in primiparous cows bred by artificial insemination

| BTA^1^ (locus) | Position (Mb)^2^ | FDR^3^ | Favorable Allele Frequency^4^ | PVE^5^ | Positional Candidate Gene(s)^6^ |
| --- | --- | --- | --- | --- | --- |
| 1 (1) | 113 | 1.50 x 10^-2^ | 0.7 | 0.026 | *-* |
| 1 (2) | 120 | 1.48 x 10^-2^ | 0.03 | 0.026 | *-* |
| 1 (2) | 120 | 1.48 x 10^-2^ | 0.97 | 0.026 | *-* |
| 1 (2) | 120 | 1.47 x 10^-2^ | 0.96 | 0.026 | *-* |
| 1 (3) | 128 | 2.48 x 10^-2^ | 0.98 | 0.027 | *-* |
| 1 (3) | 128 | 2.45 x 10^-2^ | 0.98 | 0.027 | *-* |
| 1 (3) | 128 | 2.43 x 10^-2^ | 0.98 | 0.027 | *-* |
| 1 (3) | 128 | 2.41 x 10^-2^ | 0.98 | 0.027 | *-* |
| 1 (3) | 128 | 2.39 x 10^-2^ | 0.98 | 0.027 | *-* |
| 1 (3) | 128 | 2.36 x 10^-2^ | 30.98 | 0.027 | *-* |
| 1 (3) | 128 | 2.34 x 10^-2^ | 0.98 | 0.027 | *-* |
| 1 (3) | 128 | 2.32 x 10^-2^ | 0.99 | 0.027 | *-* |
| 1 (4) | 128 | 6.34 x 10^-3^ | 0.95 | 0.032 | *-* |
| 1 (4) | 128 | 6.25 x 10^-3^ | 0.95 | 0.032 | *-* |
| 1 (4) | 128 | 6.17 x 10^-3^ | 0.95 | 0.032 | *-* |
| 1 (5) | 129 | 3.56 x 10^-4^ | 0.94 | 0.043 | *-* |
| 1 (5) | 129 | 3.29 x 10^-4^ | 0.94 | 0.043 | *-* |
| 1 (5) | 129 | 3.05 x 10^-4^ | 0.94 | 0.043 | *-* |
| 1 (5) | 129 | 2.85 x 10^-4^ | 0.94 | 0.043 | *-* |
| 1 (5) | 129 | 2.67 x 10^-4^ | 0.94 | 0.043 | *-* |
| 1 (5) | 129 | 2.51 x 10^-4^ | 0.94 | 0.043 | *-* |
| 1 (5) | 129 | 6.10 x 10^-3^ | 0.96 | 0.032 | *-* |
| 2 (6) | 4.6 | 1.49 x 10^-2^ | 0.98 | 0.026 | *AMMECR1L, POLR2D* |
| 2 (6) | 4.6 | 1.48 x 10^-2^ | 0.98 | 0.026 | *AMMECR1L, POLR2D* |
| 2 (6) | 4.6 | 1.48 x 10^-2^ | 0.98 | 0.026 | ***POLR2D****, AMMECR1L* |
| 2 (6) | 4.6 | 1.47 x 10^-2^ | 0.98 | 0.026 | ***POLR2D****, AMMECR1L* |
| 2 (6) | 4.6 | 1.47 x 10^-2^ | 0.98 | 0.026 | ***POLR2D****, AMMECR1L* |
| 2 (6) | 4.6 | 1.46 x 10^-2^ | 0.98 | 0.026 | ***POLR2D****, AMMECR1L* |
| 2 (6) | 4.6 | 1.46 x 10^-2^ | 0.98 | 0.026 | ***POLR2D****, AMMECR1L* |
| 2 (6) | 4.6 | 1.45 x 10^-2^ | 0.98 | 0.026 | *AMMECR1L, POLR2D* |
| 2 (6) | 4.6 | 1.45 x 10^-2^ | 0.98 | 0.026 | *AMMECR1L, POLR2D* |
| 2 (6) | 4.6 | 1.44 x 10^-2^ | 0.98 | 0.026 | *AMMECR1L, POLR2D* |
| 2 (6) | 4.6 | 1.44 x 10^-2^ | 0.98 | 0.026 | *AMMECR1L, POLR2D* |
| 2 (6) | 5.1 | 1.43 x 10^-2^ | 0.98 | 0.026 | *MAP3K2* |
| 2 (6) | 5.2 | 1.43 x 10^-2^ | 0.98 | 0.026 | ***MAP3K2,*** *ERCC3* |
| 2 (7) | 26 | 1.53 x 10^-2^ | 0.98 | 0.026 | *KLHL23, PHOSPHO2, CCDC173* |
| 2 (8) | 60 | 1.51 x 10^-2^ | 0.96 | 0.025 | ***THSD7B*** |
| 2 (9) | 66 | 4.30 x 10^-2^ | 0.96 | 0.023 | *-* |
| 2 (9) | 66 | 4.29 x 10^-2^ | 0.96 | 0.023 | *-* |
| 2 (9) | 66 | 4.28 x 10^-2^ | 0.96 | 0.023 | *-* |
| 3 (10) | 14 | 2.01 x 10^-2^ | 0.78 | 0.027 | ***ARHGEF2,*** *LOC112445942, RXFP4* |
| 3 (11) | 107 | 1.52 x 10^-2^ | 0.96 | 0.026 | *TRNAG-CCC* |
| 3 (11) | 108 | 1.52 x 10^-2^ | 0.99 | 0.026 | ***EPHA10,*** *CDCA8* |
| 3 (11) | 108 | 1.51 x 10^-2^ | 0.99 | 0.026 | ***CDCA8,*** *EPHA10, C3H1orf109* |
| 3 (11) | 108 | 1.51 x 10^-2^ | 0.99 | 0.026 | *CDCA8, C3H1orf109* |
| 3 (11) | 108 | 1.50 x 10^-2^ | 0.99 | 0.026 | *RSPO1* |
| 3 (12) | 110 | 5.36 x 10^-4^ | 0.98 | 0.039 | *KIAA0319L, ZMYM4* |
| 3 (12) | 110 | 1.63 x 10^-2^ | 0.91 | 0.025 | ***ZMYM4,*** *LOC112446145, LOC618220* |
| 3 (12) | 110 | 2.95 x 10^-5^ | 0.93 | 0.052 | *SFPQ* |
| 4 (13) | 13 | 7.21 x 10^-3^ | 0.98 | 0.031 | ***SLC25A13,*** *LOC112446491, LOC514680* |
| 4 (13) | 13 | 7.12 x 10^-3^ | 0.98 | 0.031 | ***SLC25A13,*** *LOC112446491, LOC514680* |
| 4 (14) | 17 | 1.54 x 10^-2^ | 0.09 | 0.026 | *-* |
| f4 (14) | 17 | 1.53 x 10^-2^ | 0.09 | 0.026 | *-* |
| 4 (14) | 17 | 1.53 x 10^-2^ | 0.09 | 0.026 | *-* |
| 4 (14) | 17 | 1.52 x 10^-2^ | 0.09 | 0.026 | *-* |
| 4 (14) | 17 | 1.51 x 10^-2^ | 0.09 | 0.026 | *-* |
| 4 (14) | 17 | 1.51 x 10^-2^ | 0.09 | 0.026 | *-* |
| 4 (14) | 17 | 1.50 x 10^-2^ | 0.09 | 0.026 | *-* |
| 4 (15) | 95 | 1.52 x 10^-2^ | 0.99 | 0.026 | ***PLXNA4*** |
| 5 (16) | 85 | 2.76 x 10^-2^ | 0.73 | 0.024 | ***SOX5*** |
| 6 (17) | 6.2 | 7.54 x 10^-3^ | 0.97 | 0.031 | ***LOC101906469, SYNPO2*** |
| 6 (17) | 6.3 | 7.77 x 10^-4^ | 0.98 | 0.040 | ***LOC101906469, SYNPO2*** |
| 6 (17) | 6.3 | 7.46 x 10^-4^ | 0.98 | 0.040 | ***LOC101906469, SYNPO2*** |
| 6 (17) | 6.3 | 6.89 x 10^-3^ | 0.98 | 0.031 | ***LOC101906469, SYNPO2*** |
| 6 (17) | 6.3 | 6.81 x 10^-3^ | 0.98 | 0.031 | ***LOC101906469, SYNPO2*** |
| 6 (17) | 6.3 | 1.60 x 10^-2^ | 0.96 | 0.025 | ***LOC101906469, SYNPO2*** |
| 6 (18) | 60 | 3.55 x 10^-2^ | 0.87 | 0.023 | ***LIMCH1,*** *LOC112447073* |
| 6 (18) | 60 | 3.55 x 10^-2^ | 0.87 | 0.023 | ***LIMCH1,*** *LOC112447073* |
| 6 (18) | 60 | 3.54 x 10^-2^ | 0.87 | 0.023 | ***LIMCH1,*** *LOC112447073* |
| 6 (18) | 61 | 1.56 x 10^-2^ | 0.85 | 0.025 | *-* |
| 7 (19) | 6.7 | 1.66 x 10^-2^ | 0.99 | 0.026 | ***AP1M1,*** *LOC112447625* |
| 7 (19) | 6.8 | 1.65 x 10^-2^ | 0.99 | 0.026 | ***AP1M1,*** *LOC112447625, FAM32A* |
| 7 (19) | 6.8 | 1.65 x 10^-2^ | 0.99 | 0.026 | ***AP1M1,*** *LOC112447625, FAM32A* |
| 7 (19) | 6.8 | 1.64 x 10^-2^ | 0.99 | 0.026 | ***AP1M1,*** *LOC112447625, FAM32A* |
| 7 (19) | 6.8 | 1.63 x 10^-2^ | 0.99 | 0.026 | ***AP1M1,*** *LOC112447625, FAM32A* |
| 7 (19) | 6.8 | 1.63 x 10^-2^ | 0.99 | 0.026 | ***AP1M1,*** *LOC112447625, FAM32A* |
| 7 (19) | 6.8 | 1.62 x 10^-2^ | 0.99 | 0.026 | ***AP1M1,*** *LOC112447625, FAM32A* |
| 7 (19) | 6.8 | 1.61 x 10^-2^ | 0.99 | 0.026 | ***AP1M1,*** *LOC112447625, FAM32A* |
| 7 (19) | 6.8 | 1.61 x 10^-2^ | 0.99 | 0.026 | ***AP1M1,*** *LOC112447625, FAM32A* |
| 7 (20) | 14 | 4.07 x 10^-2^ | 0.85 | 0.023 | ***PDE4A,*** *CDC37, LOC112447345* |
| 7 (20) | 14 | 1.92 x 10^-2^ | 0.87 | 0.028 | ***PDE4A,*** *CDC37, LOC112447345* |
| 7 (20) | 14 | 3.97 x 10^-2^ | 0.84 | 0.023 | ***PDE4A,*** *CDC37, LOC112447345* |
| 7 (21) | 46 | 1.61 x 10^-2^ | 0.93 | 0.026 | ***TXNDC15,*** *LOC112447641, PCBD2* |
| 7 (21) | 46 | 8.10 x 10^-2^ | 0.92 | 0.040 | ***PITX1,*** *CATSPER3* |
| 7 (22) | 67 | 1.62 x 10^-2^ | 0.97 | 0.025 | ***SGCD*** |
| 7 (22) | 67 | 1.61 x 10^-2^ | 0.97 | 0.025 | ***SGCD,*** *LOC616281* |
| 7 (22) | 67 | 1.61 x 10^-2^ | 0.97 | 0.025 | ***SGCD*** |
| 7 (22) | 67 | 1.61 x 10^-2^ | 0.97 | 0.025 | ***SGCD*** |
| 7 (22) | 67 | 1.60 x 10^-2^ | 0.97 | 0.025 | ***SGCD*** |
| 7 (22) | 68 | 1.61 x 10^-2^ | 0.91 | 0.025 | *-* |
| 7 (23) | 81 | 1.52 x 10^-2^ | 0.99 | 0.026 | ***SSBP2*** |
| 7 (24) | 89 | 1.51 x 10^-2^ | 0.06 | 0.026 | *-* |
| 7 (24) | 89 | 1.51 x 10^-2^ | 0.06 | 0.026 | *-* |
| 7 (24) | 89 | 1.50 x 10^-2^ | 0.06 | 0.026 | *-* |
| 7 (24) | 89 | 1.50 x 10^-2^ | 0.94 | 0.026 | *-* |
| 7 (24) | 89 | 1.50 x 10^-2^ | 0.95 | 0.026 | *-* |
| 7 (25) | 92 | 3.85 x 10^-2^ | 0.57 | 0.023 | *-* |
| 7 (25) | 92 | 3.84 x 10^-2^ | 0.57 | 0.023 | *-* |
| 8 (26) | 73 | 1.48 x 10^-2^ | 0.93 | 0.026 | ***EBF2*** |
| 8 (26) | 74 | 1.47 x 10^-2^ | 0.94 | 0.026 | ***B4GALT1,*** *SMU1* |
| 8 (26) | 74 | 1.47 x 10^-2^ | 0.94 | 0.026 | ***B4GALT1*** |
| 8 (27) | 87 | 1.50 x 10^-2^ | 0.99 | 0.026 | ***LOC112447833,*** *LOC112447927* |
| 8 (27) | 87 | 1.49 x 10^-2^ | 0.99 | 0.026 | ***LOC112447833*** |
| 8 (27) | 87 | 1.49 x 10^-2^ | 0.99 | 0.026 | ***LOC112447833*** |
| 8 (27) | 87 | 1.49 x 10^-2^ | 0.99 | 0.026 | *-* |
| 8 (28) | 104 | 1.69 x 10^-2^ | 0.61 | 0.026 | *LOC112447958* |
| 8 (29) | 106 | 1.78 x 10^-2^ | 0.5 | 0.025 | *-* |
| 8 (29) | 106 | 1.77 x 10^-2^ | 0.5 | 0.025 | *-* |
| 8 (29) | 106 | 2.11E x 10^-2^ | 0.51 | 0.025 | *-* |
| 8 (29) | 106 | 1.45 x 10^-2^ | 0.53 | 0.026 | *-* |
| 8 (29) | 106 | 1.45 x 10^-2^ | 0.53 | 0.026 | *-* |
| 8 (29) | 107 | 1.14 x 10^-2^ | 0.54 | 0.030 | *-* |
| 8 (29) | 107 | 1.13 x 10^-2^ | 0.54 | 0.030 | *TLR4* |
| 8 (29) | 107 | 1.12 x 10^-2^ | 0.54 | 0.030 | *TLR4* |
| 9 (30) | 27 | 1.56 x 10^-2^ | 0.1 | 0.026 | ***NKAIN2*** |
| 9 (30) | 28 | 1.56 x 10^-2^ | 0.89 | 0.026 | *-* |
| 9 (31) | 42 | 1.49 x 10^-2^ | 0.94 | 0.026 | *SOBP, TRNAK-CUU, PDSS2* |
| 9 (31) | 42 | 1.48 x 10^-2^ | 0.94 | 0.026 | *SOBP, TRNAK-CUU, PDSS2* |
| 9 (31) | 42 | 1.47 x 10^-2^ | 0.93 | 0.026 | ***PDSS2,*** *LOC112448046* |
| 9 (31) | 42 | 1.47 x 10^-2^ | 0.94 | 0.026 | *LOC100137843, CD24* |
| 9 (32) | 47 | 1.46 x 10^-2^ | 0.93 | 0.026 | ***LOC528043*** |
| 9 (32) | 47 | 1.46 x 10^-2^ | 0.93 | 0.026 | ***LOC528043*** |
| 9 (32) | 47 | 7.44 x 10^-2^ | 0.92 | 0.040 | ***LOC528043*** |
| 9 (32) | 47 | 1.45 x 10^-2^ | 0.93 | 0.026 | *LOC528043* |
| 9 (32) | 47 | 1.77 x 10^-2^ | 0.87 | 0.025 | *-* |
| 9 (32) | 47 | 1.76 x 10^-2^ | 0.87 | 0.025 | *-* |
| 9 (32) | 47 | 2.12 x 10^-2^ | 0.88 | 0.028 | *-* |
| 9 (32) | 47 | 2.09 x 10^-2^ | 0.88 | 0.028 | *-* |
| 9 (32) | 47 | 2.07 x 10^-2^ | 0.88 | 0.028 | *-* |
| 9 (32) | 47 | 6.38 x 10^-3^ | 0.89 | 0.032 | *-* |
| 9 (32) | 47 | 6.30 x 10^-3^ | 0.89 | 0.032 | *-* |
| 9 (32) | 47 | 6.21 x 10^-3^ | 0.89 | 0.032 | *-* |
| 9 (32) | 47 | 2.26 x 10^-2^ | 0.85 | 0.028 | *-* |
| 9 (32) | 47 | 2.24 x 10^-2^ | 0.85 | 0.028 | *-* |
| 9 (32) | 47 | 2.21 x 10^-2^ | 0.85 | 0.028 | *-* |
| 9 (32) | 47 | 2.19 x 10^-2^ | 0.85 | 0.028 | *-* |
| 9 (32) | 47 | 4.27 x 10^-2^ | 0.86 | 0.023 | *-* |
| 9 (32) | 47 | 4.26 x 10^-2^ | 0.86 | 0.023 | *-* |
| 9 (33) | 90 | 1.50 x 10^-2^ | 0.94 | 0.026 | ***OPRM1,*** *IPCEF1* |
| 9 (33) | 90 | 1.50 x 10^-2^ | 0.94 | 0.026 | ***OPRM1,*** *IPCEF1* |
| 10 (34) | 13 | 1.73 x 10^-2^ | 0.96 | 0.027 | ***LOC112441475*** |
| 10 (34) | 13 | 1.72 x 10^-2^ | 0.96 | 0.027 | ***LOC112441475*** |
| 10 (34) | 13 | 1.71 x 10^-2^ | 0.96 | 0.027 | ***LOC112441475*** |
| 10 (34) | 13 | 1.70 x 10^-2^ | 0.96 | 0.027 | *LOC112441475* |
| 10 (34) | 13 | 1.69 x 10^-2^ | 0.96 | 0.027 | *LOC112441475* |
| 10 (34) | 13 | 1.68 x 10^-2^ | 0.96 | 0.027 | *SMAD6* |
| 10 (35) | 95 | 1.71 x 10^-2^ | 0.96 | 0.027 | *-* |
| 10 (35) | 95 | 1.70 x 10^-2^ | 0.96 | 0.027 | *-* |
| 10 (35) | 95 | 1.69 x 10^-2^ | 0.96 | 0.027 | *-* |
| 10 (35) | 95 | 1.68 x 10^-2^ | 0.96 | 0.027 | *-* |
| 10 (35) | 98 | 2.06 x 10^-2^ | 0.92 | 0.027 | *-* |
| 12 (36) | 52 | 5.04 x 10^-4^ | 0.98 | 0.039 | *LOC112449132* |
| 12 (37) | 52 | 4.95 x 10^-4^ | 0.98 | 0.039 | *LOC112449132* |
| 12 (37) | 52 | 4.86 x 10^-4^ | 0.98 | 0.039 | *LOC112449132* |
| 12 (37) | 52 | 4.77 x 10^-4^ | 0.99 | 0.039 | *LOC112449132* |
| 12 (37) | 52 | 4.69 x 10^-4^ | 0.99 | 0.039 | *-* |
| 12 (37) | 78 | 4.24 x 10^-2^ | 0.82 | 0.023 | *TPP2, METTL21C, CCDC168* |
| 12 (38) | 78 | 4.23 x 10^-2^ | 0.82 | 0.023 | *METTL21C, CCDC168* |
| 12 (38) | 78 | 4.22 x 10^-2^ | 0.82 | 0.023 | *METTL21C, CCDC168* |
| 12 (38) | 78 | 4.21 x 10^-2^ | 0.82 | 0.023 | *METTL21C, CCDC168* |
| 13 (38) | 6.1 | 1.56 x 10^-2^ | 0.94 | 0.026 | *-* |
| 13 (39) | 6.1 | 1.56 x 10^-2^ | 0.94 | 0.026 | *-* |
| 13 (39) | 75 | 2.24 x 10^-2^ | 0.98 | 0.027 | ***ELMO2,*** *SLC35C2* |
| 13 (40) | 75 | 2.22 x 10^-2^ | 0.95 | 0.027 | *-* |
| 13 (40) | 75 | 2.20 x 10^-2^ | 0.95 | 0.027 | *-* |
| 13 (40) | 75 | 2.18 x 10^-2^ | 0.95 | 0.027 | *-* |
| 13 (40) | 75 | 2.16 x 10^-2^ | 0.95 | 0.027 | *-* |
| 13 (40) | 75 | 2.15 x 10^-2^ | 0.95 | 0.027 | *-* |
| 13 (40) | 75 | 2.13 x 10^-2^ | 0.95 | 0.027 | *LOC100847115* |
| 13 (40) | 75 | 2.11 x 10^-2^ | 0.95 | 0.027 | *LOC100847115* |
| 13 (40) | 75 | 2.09 x 10^-2^ | 0.95 | 0.027 | *LOC100847115, LOC101904842* |
| 13 (40) | 75 | 2.08 x 10^-2^ | 0.95 | 0.027 | *LOC100847115, LOC101904842* |
| 13 (40) | 75 | 5.37 x 10^-4^ | 0.98 | 0.039 | ***SLC13A3,*** *TRNAR-CCU* |
| 13 (41) | 75 | 5.25 x 10^-4^ | 0.99 | 0.039 | ***SLC13A3*** |
| 13 (41) | 75 | 5.15 x 10^-4^ | 0.99 | 0.039 | ***SLC13A3*** |
| 13 (41) | 75 | 5.04 x 10^-4^ | 0.99 | 0.039 | ***SLC13A3,*** *LOC112449242, TP53RK* |
| 13 (41) | 75 | 1.59 x 10^-2^ | 0.98 | 0.025 | ***EYA2*** |
| 13 (41) | 75 | 4.94 x 10^-4^ | 0.95 | 0.039 | ***EYA2*** |
| 13 (41) | 75 | 2.08 x 10^-2^ | 0.98 | 0.027 | ***EYA2*** |
| 13 (41) | 75 | 2.06 x 10^-2^ | 0.98 | 0.027 | ***EYA2*** |
| 13 (41) | 75 | 2.05 x 10^-2^ | 0.98 | 0.027 | ***EYA2*** |
| 13 (41) | 75 | 2.03 x 10^-2^ | 0.98 | 0.027 | ***EYA2*** |
| 13 (41) | 75 | 2.02 x 10^-2^ | 0.98 | 0.027 | ***EYA2*** |
| 13 (41) | 75 | 4.85 x 10^-4^ | 0.98 | 0.039 | ***EYA2*** |
| 13 (41) | 75 | 2.31 x 10^-2^ | 0.71 | 0.024 | ***ZMYND8,*** *LOC101905203, LOC112449409* |
| 13 (41) | 75 | 2.07 x 10^-2^ | 0.72 | 0.027 | ***ZMYND8,*** *LOC104973934* |
| 14 (41) | 22 | 1.76 x 10^-2^ | 0.91 | 0.026 | *RP1* |
| 14 (41) | 55 | 4.61 x 10^-4^ | 0.98 | 0.039 | *-* |
| 14 (42) | 55 | 4.53 x 10^-4^ | 0.98 | 0.039 | *-* |
| 14 (43) | 64 | 1.45 x 10^-2^ | 0.98 | 0.026 | ***VPS13B*** |
| 14 (43) | 64 | 1.44 x 10^-2^ | 0.98 | 0.026 | ***VPS13B*** |
| 14 (44) | 65 | 1.44 x 10^-2^ | 0.98 | 0.026 | ***VPS13B*** |
| 14 (44) | 67 | 1.93 x 10^-2^ | 0.95 | 0.027 | ***CPQ*** |
| 14 (44) | 67 | 1.91 x 10^-2^ | 0.95 | 0.027 | ***CPQ*** |
| 14 (45) | 67 | 1.90 x 10^-2^ | 0.95 | 0.027 | ***CPQ*** |
| 14 (45) | 67 | 1.89 x 10^-2^ | 0.95 | 0.027 | ***CPQ*** |
| 14 (45) | 67 | 1.87 x 10^-2^ | 0.95 | 0.027 | ***CPQ*** |
| 14 (45) | 67 | 1.86 x 10^-2^ | 0.95 | 0.027 | ***CPQ*** |
| 14 (45) | 67 | 1.85 x 10^-2^ | 0.95 | 0.027 | ***CPQ*** |
| 16 (45) | 16 | 1.72 x 10^-3^ | 0.7 | 0.035 | *-* |
| 16 (45) | 16 | 1.69 x 10^-3^ | 0.7 | 0.035 | *-* |
| 16 (46) | 16 | 4.88 x 10^-4^ | 0.86 | 0.039 | *-* |
| 16 (46) | 16 | 1.43 x 10^-3^ | 0.84 | 0.036 | *-* |
| 16 (46) | 16 | 1.41 x 10^-3^ | 0.84 | 0.036 | *-* |
| 16 (46) | 16 | 1.38 x 10^-3^ | 0.84 | 0.036 | *-* |
| 16 (46) | 16 | 1.36 x 10^-3^ | 0.84 | 0.036 | *-* |
| 16 (46) | 53 | 1.61 x 10^-2^ | 0.98 | 0.026 | *-* |
| 16 (46) | 53 | 7.20 x 10^-4^ | 0.94 | 0.040 | *-* |
| 16 (47) | 53 | 6.95 x 10^-4^ | 0.94 | 0.040 | *-* |
| 17 (47) | 36 | 3.50 x 10^-2^ | 0.55 | 0.023 | ***FSTL5*** |
| 17 (47) | 36 | 1.47 x 10^-2^ | 0.99 | 0.026 | *-* |
| 17 (48)**^*^** | 51 | 4.91 x 10^-2^ | 0.94 | 0.037 | ***NCOR2*** |
| 17 (49) | 54 | 8.22 x 10^-4^ | 0.99 | 0.039 | *-* |
| 17 (50) | 54 | 7.95 x 10^-4^ | 0.99 | 0.039 | *CUX2* |
| 17 (51) | 54 | 7.69 x 10^-4^ | 0.99 | 0.039 | *CUX2* |
| 17 (51) | 54 | 7.45 x 10^-4^ | 0.99 | 0.039 | *CUX2* |
| 17 (51) | 54 | 7.22 x 10^-4^ | 0.96 | 0.039 | ***CUX2*** |
| 17 (51) | 54 | 7.01 x 10^-4^ | 0.98 | 0.039 | ***CUX2*** |
| 17 (51) | 55 | 6.81 x 10^-4^ | 0.99 | 0.039 | ***CUX2,*** *PHETA1* |
| 17 (51) | 55 | 6.62 x 10^-4^ | 0.99 | 0.039 | ***CUX2,*** *PHETA1* |
| 17 (51) | 55 | 6.44 x 10^-4^ | 0.99 | 0.039 | ***CUX2,*** *PHETA1* |
| 17 (51) | 55 | 6.27 x 10^-4^ | 0.99 | 0.039 | ***CUX2,*** *PHETA1* |
| 17 (51) | 55 | 6.11 x 10^-4^ | 0.99 | 0.039 | ***CUX2,*** *PHETA1* |
| 17 (51) | 55 | 5.96 x 10^-4^ | 0.99 | 0.039 | ***CUX2,*** *PHETA1* |
| 17 (51) | 55 | 5.82 x 10^-4^ | 0.99 | 0.039 | ***CUX2,*** *PHETA1* |
| 17 (51) | 55 | 5.68 x 10^-4^ | 0.99 | 0.039 | *CUX2, PHETA1* |
| 17 (51) | 55 | 5.54 x 10^-4^ | 0.99 | 0.039 | *CUX2, PHETA1* |
| 17 (51) | 55 | 5.42 x 10^-4^ | 0.98 | 0.039 | ***SH2B3,*** *ATXN2* |
| 17 (51) | 55 | 5.30 x 10^-4^ | 0.99 | 0.039 | ***BRAP,*** *LOC513508* |
| 17 (52) | 60 | 5.77 x 10^-3^ | 0.89 | 0.032 | *LOC100848596, LOC107133302* |
| 17 (52) | 60 | 5.68 x 10^-3^ | 0.89 | 0.032 | *LOC100848596, LOC107133302* |
| 17 (53) | 64 | 2.93 x 10^-2^ | 0.88 | 0.024 | ***SVOP,*** *DAO* |
| 17 (54) | 65 | 1.70 x 10^-2^ | 0.9 | 0.026 | ***KIAA1671,*** *CRYBB3, CRYBB2* |
| 17 (54) | 65 | 1.69 x 10^-2^ | 0.9 | 0.026 | ***KIAA1671,*** *CRYBB3, CRYBB2* |
| 17 (54) | 65 | 1.68 x 10^-2^ | 0.9 | 0.026 | ***CRYBB3,*** *KIAA1671, CRYBB2* |
| 17 (54) | 65 | 1.68 x 10^-2^ | 0.9 | 0.026 | ***CRYBB3,*** *KIAA1671, CRYBB2* |
| 17 (54) | 65 | 1.67 x 10^-2^ | 0.9 | 0.026 | ***CRYBB3,*** *KIAA1671, CRYBB2* |
| 17 (54) | 65 | 1.66 x 10^-2^ | 0.9 | 0.026 | ***CRYBB3,*** *KIAA1671, CRYBB2* |
| 17 (55) | 65 | 2.24 x 10^-3^ | 0.87 | 0.034 | *LOC112442007, GRK3* |
| 18 (56) | 9.1 | 1.49 x 10^-2^ | 0.9 | 0.026 | *CDH13* |
| 18 (56) | 9.1 | 1.49 x 10^-2^ | 0.9 | 0.026 | *-* |
| 18 (57) | 18 | 2.02 x 10^-2^ | 0.91 | 0.028 | *PAPD5, LOC112442434, LOC112442433, ADCY7* |
| 18 (58) | 23 | 6.28 x 10^-3^ | 0.93 | 0.032 | *LOC785669* |
| 18 (59) | 33 | 1.61 x 10^-2^ | 0.94 | 0.026 | *-* |
| 18 (60) | 41 | 2.84 x 10^-2^ | 0.59 | 0.024 | ***ZNF536*** |
| 18 (60) | 41 | 1.62 x 10^-2^ | 0.59 | 0.025 | ***ZNF536*** |
| 18 (61) | 45 | 1.84 x 10^-2^ | 0.93 | 0.028 | *LOC789050, ZNF792* |
| 18 (61) | 45 | 1.82 x 10^-2^ | 0.93 | 0.028 | *ZNF792, LOC100297240, GRAMD1A* |
| 20 (62) | 6.3 | 1.83 x 10^-2^ | 0.96 | 0.026 | *MIR584-6* |
| 20 (62) | 6.3 | 1.82 x 10^-2^ | 0.96 | 0.026 | *MIR584-6* |
| 20 (62) | 6.3 | 1.82 x 10^-2^ | 0.96 | 0.026 | *MIR584-6* |
| 20 (62) | 6.3 | 2.26 x 10^-2^ | 0.94 | 0.028 | *-* |
| 20 (62) | 6.4 | 2.24 x 10^-2^ | 0.96 | 0.028 | *MSX2* |
| 20 (62) | 6.4 | 1.81 x 10^-2^ | 0.96 | 0.026 | *-* |
| 20 (62) | 6.5 | 1.80 x 10^-2^ | 0.96 | 0.026 | *-* |
| 20 (62) | 6.5 | 1.79 x 10^-2^ | 0.96 | 0.026 | *-* |
| 20 (62) | 6.6 | 1.78 x 10^-2^ | 0.96 | 0.026 | *-* |
| 20 (62) | 6.6 | 1.77 x 10^-2^ | 0.96 | 0.026 | *-* |
| 20 (62) | 6.7 | 1.77 x 10^-2^ | 0.96 | 0.026 | ***FAM169A,*** *NSA2, GFM2* |
| 20 (62) | 6.7 | 1.76 x 10^-2^ | 0.96 | 0.026 | ***GFM2,*** *FAM169A, NSA2* |
| 20 (62) | 6.7 | 1.75 x 10^-2^ | 0.96 | 0.026 | ***GFM2,*** *FAM169A, NSA2* |
| 20 (62) | 6.7 | 1.74 x 10^-2^ | 0.96 | 0.026 | ***GFM2,*** *FAM169A, NSA2* |
| 20 (62) | 6.7 | 1.73 x 10^-2^ | 0.96 | 0.026 | ***GFM2,*** *HEXB* |
| 20 (62) | 6.8 | 1.73 x 10^-2^ | 0.96 | 0.026 | *GFM2, HEXB, LOC786974* |
| 20 (62) | 6.8 | 1.72 x 10^-2^ | 0.96 | 0.026 | ***LOC786974,*** *GFM2, HEXB, LOC104975198* |
| 20 (62) | 6.8 | 1.71 x 10^-2^ | 0.95 | 0.026 | ***LOC104975198,*** *LOC786974* |
| 20 (62) | 6.8 | 1.70 x 10^-2^ | 0.95 | 0.026 | *LOC786974, LOC104975198* |
| 20 (62) | 6.8 | 1.70 x 10^-2^ | 0.96 | 0.026 | *LOC786974, LOC104975198, ENC1* |
| 20 (62) | 6.8 | 1.69 x 10^-2^ | 0.96 | 0.026 | *LOC786974, LOC104975198, ENC1* |
| 20 (62) | 6.9 | 2.22 x 10^-2^ | 0.95 | 0.028 | ***ENC1*** |
| 20 (62) | 6.9 | 2.19 x 10^-2^ | 0.95 | 0.028 | *ENC1* |
| 20 (62) | 6.9 | 1.68 x 10^-2^ | 0.96 | 0.026 | *-* |
| 20 (62) | 6.9 | 1.67 x 10^-2^ | 0.96 | 0.026 | *-* |
| 20 (63) | 11 | 1.45 x 10^-2^ | 0.98 | 0.026 | ***LOC104975214*** |
| 20 (64) | 16 | 2.15 x 10^-2^ | 0.94 | 0.027 | *-* |
| 20 (64) | 16 | 2.13 x 10^-2^ | 0.94 | 0.027 | *-* |
| 20 (64) | 16 | 2.12 x 10^-2^ | 0.94 | 0.027 | *-* |
| 20 (64) | 16 | 2.11 x 10^-2^ | 0.94 | 0.027 | *-* |
| 20 (64) | 16 | 2.09 x 10^-2^ | 0.94 | 0.027 | *-* |
| 20 (64) | 16 | 2.08 x 10^-2^ | 0.94 | 0.027 | *-* |
| 20 (64) | 16 | 2.07 x 10^-2^ | 0.94 | 0.027 | *-* |
| 20 (64) | 16 | 2.05 x 10^-2^ | 0.94 | 0.027 | *-* |
| 20 (64) | 16 | 2.04 x 10^-2^ | 0.94 | 0.027 | *-* |
| 20 (64) | 16 | 2.03 x 10^-2^ | 0.94 | 0.027 | *-* |
| 20 (64) | 16 | 2.02 x 10^-2^ | 0.94 | 0.027 | *-* |
| 20 (64) | 16 | 2.00 x 10^-2^ | 0.94 | 0.027 | *-* |
| 20 (64) | 16 | 1.99 x 10^-2^ | 0.94 | 0.027 | *-* |
| 20 (64) | 16 | 1.98 x 10^-2^ | 0.94 | 0.027 | *-* |
| 20 (64) | 16 | 1.97 x 10^-2^ | 0.94 | 0.027 | *-* |
| 20 (64) | 16 | 1.96 x 10^-2^ | 0.94 | 0.027 | *-* |
| 20 (64) | 16 | 1.95 x 10^-2^ | 0.94 | 0.027 | *-* |
| 20 (64) | 16 | 1.93 x 10^-2^ | 0.94 | 0.027 | *-* |
| 20 (64) | 16 | 1.92 x 10^-2^ | 0.94 | 0.027 | *-* |
| 20 (64) | 16 | 1.91 x 10^-2^ | 0.94 | 0.027 | *-* |
| 20 (64) | 16 | 1.90 x 10^-2^ | 0.94 | 0.027 | *-* |
| 20 (64) | 16 | 1.89 x 10^-2^ | 0.94 | 0.027 | *-* |
| 20 (64) | 16 | 1.88 x 10^-2^ | 0.94 | 0.027 | *-* |
| 20 (64) | 16 | 1.87 x 10^-2^ | 0.94 | 0.027 | *-* |
| 20 (64) | 16 | 1.86 x 10^-2^ | 0.94 | 0.027 | *-* |
| 20 (64) | 16 | 1.85 x 10^-2^ | 0.94 | 0.027 | *-* |
| 20 (64) | 16 | 1.84 x 10^-2^ | 0.94 | 0.027 | *-* |
| 20 (64) | 16 | 1.83 x 10^-2^ | 0.94 | 0.027 | *-* |
| 20 (64) | 16 | 1.82 x 10^-2^ | 0.94 | 0.027 |  |
| 20 (64) | 16 | 1.81 x 10^-2^ | 0.94 | 0.027 | *-* |
| 20 (64) | 16 | 1.80 x 10^-2^ | 0.94 | 0.027 | *-* |
| 20 (64) | 16 | 1.79 x 10^-2^ | 0.94 | 0.027 | *-* |
| 20 (64) | 16 | 1.78 x 10^-2^ | 0.94 | 0.027 | *-* |
| 20 (64) | 16 | 1.7 x 10^-2^ | 0.94 | 0.027 | *-* |
| 20 (64) | 16 | 1.76 x 10^-2^ | 0.94 | 0.027 | *-* |
| 20 (64) | 16 | 1.75 x 10^-2^ | 0.94 | 0.027 | *-* |
| 20 (64) | 16 | 1.74 x 10^-2^ | 0.94 | 0.027 | *-* |
| 20 (64) | 16 | 1.73 x 10^-2^ | 0.94 | 0.027 | *-* |
| 20 (64) | 16 | 1.72 x 10^-2^ | 0.94 | 0.027 | *-* |
| 20 (64) | 16 | 1.71 x 10^-2^ | 0.94 | 0.027 | *-* |
| 20 (64) | 16 | 1.70 x 10^-2^ | 0.94 | 0.027 | *-* |
| 20 (64) | 16 | 1.70 x 10^-2^ | 0.94 | 0.027 | *-* |
| 20 (64) | 16 | 1.69 x 10^-2^ | 0.94 | 0.027 | *-* |
| 20 (64) | 16 | 1.68 x 10^-2^ | 0.94 | 0.027 | *-* |
| 20 (64) | 16 | 1.67 x 10^-2^ | 0.94 | 0.027 | *-* |
| 22 (65) | 1.9 | 1.66 x 10^-2^ | 0.94 | 0.053 | *-* |
| 22 (66) | 3.9 | 4.07 x 10^-5^ | 0.95 | 0.028 | ***RBMS3*** |
| 23 (67) | 5.5 | 2.33 x 10^-2^ | 0.87 | 0.025 | ***FAM83B*** |
| 23 (67) | 5.5 | 1.98 x 10^-2^ | 0.71 | 0.025 | ***FAM83B*** |
| 23 (68) | 42 | 1.97 x 10^-2^ | 0.71 | 0.026 | ***RANBP9****, NOL7, SIRT5* |
| 23 (68) | 43 | 1.57 x 10^-2^ | 0.93 | 0.026 | ***RANBP9,*** *NOL7, SIRT5* |
| 23 (68) | 43 | 1.57 x 10^-2^ | 0.93 | 0.026 | ***RANBP9,*** *NOL7, SIRT5* |
| 23 (68) | 43 | 1.56 x 10^-2^ | 0.93 | 0.026 | ***PHACTR1*** |
| 23 (68) | 43 | 1.55 x 10^-2^ | 0.92 | 0.026 | ***PHACTR1*** |
| 23 (68) | 43 | 1.55 x 10^-2^ | 0.92 | 0.026 | ***PHACTR1*** |
| 23 (68) | 44 | 1.54 x 10^-2^ | 0.92 | 0.026 | *-* |
| 23 (68) | 44 | 1.54 x 10^-2^ | 0.93 | 0.026 | *-* |
| 24 (69) | 23 | 1.5 x 10^-2^ | 0.93 | 0.026 | *NOL4* |
| 24 (69) | 23 | 1.46 x 10^-2^ | 0.98 | 0.026 | *NOL4* |
| 24 (69) | 23 | 1.46 x 10^-2^ | 0.98 | 0.026 | *NOL4* |
| 24 (69) | 23 | 1.45 x 10^-2^ | 0.98 | 0.026 | *NOL4* |
| 24 (69) | 23 | 1.45 x 10^-2^ | 0.98 | 0.026 | *NOL4* |
| 24 (69) | 23 | 1.45 x 10^-2^ | 0.98 | 0.027 | ***ASXL3*** |
| 24 (69) | 23 | 1.95 x 10^-2^ | 0.98 | 0.026 | ***ASXL3*** |
| 24 (69) | 23 | 1.44 x 10^-2^ | 0.98 | 0.026 | ***ASXL3*** |
| 24 (69) | 23 | 1.44 x 10^-2^ | 0.98 | 0.026 | ***ASXL3*** |
| 24 (69) | 23 | 1.43 x 10^-2^ | 0.98 | 0.026 | ***ASXL3*** |
| 24 (69) | 23 | 1.43 x 10^-2^ | 0.98 | 0.026 | ***ASXL3*** |
| 24 (70) | 34 | 1.42 x 10^-2^ | 0.98 | 0.027 | *GATA6, LOC100336909* |
| 24 (70) | 34 | 2.31 x 10^-2^ | 0.97 | 0.027 | *-* |
| 24 (70) | 34 | 2.29 x 10^-2^ | 0.97 | 0.027 | *-* |
| 24 (70) | 34 | 2.27 x 10^-2^ | 0.97 | 0.027 | *-* |
| 24 (70) | 34 | 2.25 x 10^-2^ | 0.97 | 0.027 | *-* |
| 24 (71) | 35 | 2.23 x 10^-2^ | 0.97 | 0.043 | ***COLEC12*** |
| 24 (71) | 35 | 2.99 x 10^-4^ | 0.88 | 0.043 | *CETN1, LOC112444249, CLUL1* |
| 24 (71) | 35 | 2.84 x 10^-4^ | 0.87 | 0.043 | *CETN1, LOC112444249, CLUL1* |
| 24 (71) | 35 | 2.70 x 10^-4^ | 0.87 | 0.043 | ***CLUL1****, CETN1, LOC112444249* |
| 24 (71) | 35 | 2.57 x 10^-4^ | 0.87 | 0.043 | ***CLUL1,*** *LOC112444249, TYMS* |
| 24 (72) | 35 | 2.45 x 10^-4^ | 0.87 | 0.023 | *CLUL1, TYMS, ENOSF1, LOC112444211* |
| 24 (73) | 35 | 4.30 x 10^-2^ | 0.89 | 0.053 | *LOC786055* |
| 24 (74) | 37 | 3.52 x 10^-5^ | 0.91 | 0.026 | ***DLGAP1,*** *LOC112444209* |
| 24 (75) | 39 | 1.41 x 10^-2^ | 0.98 | 0.026 | *ARHGAP28* |
| 24 (75) | 39 | 1.45 x 10^-2^ | 0.93 | 0.026 | ***ARHGAP28*** |
| 24 (75) | 39 | 1.45 x 10^-2^ | 0.93 | 0.026 | ***ARHGAP28*** |
| 24 (75) | 39 | 1.45 x 10^-2^ | 0.93 | 0.026 | ***ARHGAP28*** |
| 24 (75) | 39 | 1.44 x 10^-2^ | 0.93 | 0.026 | ***ARHGAP28*** |
| 24 (75) | 39 | 1.44 x 10^-2^ | 0.93 | 0.026 | ***ARHGAP28*** |
| 24 (75) | 39 | 1.43 x 10^-2^ | 0.93 | 0.026 | ***ARHGAP28*** |
| 24 (75) | 39 | 1.43 x 10^-2^ | 0.93 | 0.026 | ***ARHGAP28*** |
| 24 (75) | 39 | 1.43 x 10^-2^ | 0.93 | 0.026 | ***ARHGAP28*** |
| 24 (75) | 40 | 1.42 x 10^-2^ | 0.93 | 0.026 | ***LAMA1*** |
| 25 (76) | 14 | 1.42 x 10^-2^ | 0.93 | 0.026 | *-* |
| 25 (76) | 14 | 1.52 x 10^-2^ | 0.88 | 0.026 | *-* |
| 25 (77) | 17 | 1.52 x 10^-2^ | 0.88 | 0.027 | ***IQCK,*** *GPRC5B* |
| 26 (78) | 31 | 1.92 x 10^-2^ | 0.99 | 0.029 | *-* |
| 26 (78) | 31 | 1.43 x 10^-2^ | 0.7 | 0.027 | *-* |
| 26 (78) | 31 | 2.07 x 10^-2^ | 0.69 | 0.024 | *-* |
| 26 (79) | 36 | 3.04 x 10^-2^ | 0.68 | 0.026 | ***ATRNL1*** |
| 26 (79) | 36 | 1.45 x 10^-2^ | 0.93 | 0.026 | ***ATRNL1*** |
| 26 (79) | 36 | 1.44 x 10^-2^ | 0.93 | 0.026 | *ATRNL1* |
| 28 (80) | 7.6 | 1.44 x 10^-2^ | 0.93 | 0.026 | *-* |
| 28 (80) | 7.6 | 1.42 x 10^-2^ | 0.94 | 0.026 | *-* |
| 28 (80) | 7.6 | 1.42 x 10^-2^ | 0.94 | 0.026 | *-* |
| 28 (81) | 9.4 | 1.41 x 10^-2^ | 0.94 | 0.043 | ***MTR*** |
| 28 (81) | 9.4 | 6.83 x 10^-4^ | 0.94 | 0.043 | ***MTR*** |
| 28 (81) | 9.4 | 5.85 x 10^-4^ | 0.94 | 0.043 | ***MTR*** |
| 28 (81) | 9.4 | 5.12 x 10^-4^ | 0.93 | 0.043 | ***MTR*** |
| 28 (81) | 9.4 | 4.55 x 10^-4^ | 0.93 | 0.043 | ***MTR*** |
| 28 (81) | 9.4 | 4.10 x 10^-4^ | 0.93 | 0.043 | ***MTR*** |
| 28 (81) | 9.4 | 3.72 x 10^-4^ | 0.93 | 0.048 | ***MTR*** |
| 28 (81) | 9.5 | 1.46 x 10^-4^ | 0.93 | 0.056 | ***MTR,*** *TRNAE-UUC* |
| 28 (82) | 39 | 3.07 x 10^-5^ | 0.93 | 0.026 | *LOC107131937* |
| 29 (83) | 48 | 1.43 x 10^-2^ | 0.89 | 0.027 | ***KCNQ1*** |
| 29 (83) | 48 | 2.01 x 10^-2^ | 0.87 | 0.027 | ***KCNQ1*** |
| 29 (83) | 48 | 2.00 x 10^-2^ | 0.87 | 0.027 | ***KCNQ1*** |
| 29 (83) | 48 | 1.98 x 10^-2^ | 0.87 | 0.027 | ***KCNQ1*** |
| 29 (83) | 48 | 1.97 x 10^-2^ | 0.87 | 0.027 | ***KCNQ1*** |
| 29 (83) | 48 | 1.96 x 10^-2^ | 0.87 | 0.027 | ***KCNQ1,*** *LOC112444897* |
| 29 (83) | 48 | 1.94 x 10^-2^ | 0.87 | 0.027 | ***KCNQ1,*** *LOC112444897* |
| 29 (83) | 48 | 1.93 x 10^-2^ | 0.87 | 0.024 | ***KCNQ1,*** *LOC112444897* |
| X (84) | 71 | 2.85 x 10^-2^ | 0.87 | 0.034 | ***SH3BGRL*** |
| X (84) | 71 | 2.38 x 10^-3^ | 0.92 | 0.034 | *-* |
| X (85) | 126 | 2.35 x 10^-3^ | 0.92 | 0.025 | ***REPS2*** |
| X (85) | 126 | 1.83 x 10^-2^ | 0.77 | 0.025 | ***RBBP7,*** *TXLNG* |
| X (85) | 126 | 1.82 x 10^-2^ | 0.77 | 0.025 | ***TXLNG,*** *RBBP7* |
| X (85) | 126 | 1.82 x 10^-2^ | 0.77 | 0.025 | ***TXLNG,*** *RBBP7* |
| X (85) | 126 | 1.81 x 10^-2^ | 0.77 | 0.025 | ***SYAP1,*** *LOC112445211, CTPS2* |
| X (85) | 127 | 1.81 x 10^-2^ | 0.77 | 0.024 | *-* |
| X (85) | 127 | 2.85 x 10^-2^ | 0.72 | 0.023 | *-* |
| X (85) | 127 | 3.84 x 10^-2^ | 0.71 | 0.022 | *-* |
| X (85) | 127 | 1.61 x 10^-2^ | 0.94 | 0.022 | *-* |
| X (86) | 137 | 1.60 x 10^-2^ | 0.94 | 0.026 | *LOC505052, LOC101904044, LOC104970061* |
| X (86) | 137 | 1.59 x 10^-2^ | 0.94 | 0.026 | *LOC505052, LOC101904044, LOC104970061* |
| X (86) | 137 | 1.59 x 10^-2^ | 0.94 | 0.026 | *LOC101904044, LOC104970061* |
| X (86) | 137 | 1.58 x 10^-2^ | 0.93 | 0.026 | ***OBP,*** *LOC112445027* |
| X (86) | 137 | 1.57 x 10^-2^ | 0.87 | 0.026 | *LOC112445112, LOC104969973, LOC104970733, LOC104969972, LOC100847998* |
| X (86) | 137 | 1.91 x 10^-2^ | 0.88 | 0.027 | *LOC104969973, LOC104970733, LOC104969972, LOC 100847998* |
| X (86) | 137 | 1.57 x 10^-2^ | 0.93 | 0.026 | *LOC100847998* |

^1^*Bos taurus* chromosome. In parentheses are the sequentially numbered loci from BTA1 to BTAX that are associated with spontaneous and the corresponding associated single nucleotide polymorphisms (SNPs). ^2^Location of associated SNPs, in Megabases (Mb), measured by the numbered nucleotides in the ARS-UCD 1.2 reference genome assembly (https://www.ncbi.nlm.nih.gov/datasets/genome/GCF_002263795.1/). ^3^FDR corrected *P*-value for each SNP associated with spontaneous abortion. ^4^Favorable allele frequency within the population. ^5^The proportion of variance explained (PVE) for each SNP associated with spontaneous abortion. ^6^Positional candidate genes located within 30.5 Kb on either side of the associated SNP(s) within each locus. **Bolded** gene names represent genes where the SNP is located within the gene. ^*^Locus associated in the additive inheritance model

**Table S2**: Positional candidate genes associated with spontaneous abortion in primiparous cows bred by artificial insemination that share common functions.

| **Common Function^1^** | **# Genes^2^** | **Positional Candidate Genes^3^** |
| --- | --- | --- |
| Regulation of translation | 3 | *ATXN2, GFM2, MIR584-6* |
| Transfer RNA | 4 | *TRNAE-UUC, TRNAG-CCC, TRNAK-CUU, TRNAR-CCU* |
| G Protein-coupled receptors/receptor activity | 5 | *ARHGEF2, ATRNL1, GPR149, GRK3, RXFP4* |
| Response to stress | 6 | *CLUL1, ENC1, IPCEF1, LIMCH1, SFPQ, SIRT5* |
| Ion transport, calcium binding, calmodulin binding | 7 | *CATSPER3, CLSTN2, FSTL5, IQCK, KCNQ1, LOC100297240, NKAIN2* |
| Immune tesponse | 7 | *CD24, COLEC12, KIAA0319L, SH2B3, TLR4, TRIM42, ZMYND8* |
| Nervous system - voltage gated channels, neurotransmitter release, neuronal responses, function as receptors in nervous system | 9 | *CDH13, CLSTN2, DLGAP1, EPHA10, HEXB, KCNQ1, PLXNA4, VPS13B, ZNF536* |
| Cell cycle, DNA replication, mitosis/meiosis | 10 | *CDC37, CDCA8, CETN1, LAMA1, LIMCH1, MEGF10, NSA2, RBMS3, TXLNG, TYMS* |
| Apoptosis signaling | 10 | *CDH13, ELMO2, FAM32A, MEGF10, MSX2, RBMS3, RP1, SFPQ, TP53RK, TRIM42* |
| Transport of molecules between cells and within a cell | 11 | *AP1M1, ATXN2, BRAP, CATSPER3, KCNQ1, NKAIN2, PHACTR1, RANBP9, REPS2, SLC13A3, VPS13B* |
| Regulation of alternative splicing/small nucleolar RNA - critical components of the spliceosome | 13 | *LOC112442433, LOC112442434, LOC112444249, LOC112445211, LOC112446145, LOC112446491, LOC112447625, LOC112447927, LOC112447958, LOC112448327, LOC112449409, SFPQ, SMU1* |
| Metabolic pathways - protein, carbohydrates, ATP | 14 | *ADCY7, ASXL3, CPQ, DLGAP1, ENOSF1, GPRC5B, GRAMD1A, MTR, PAPD5, PHOSPHO2, SIRT5, SLC13A3, TP53RK, TPP2* |
| Signal transduction and signaling pathways | 15 | *ARHGAP28, ATRNL1, CDC37, FAM83B, GRK3, MAP3K2, MSX2, PDE4A, RSPO1, SH2B3, SMAD6, SYNPO2, TP53RK, TRIM42, ZMYND8* |
| Transcription factor activity; transcription factor; regulation RNA polymerase II regulation; transcription coregulatory activity; regulation of transcription | 16 | *ASXL3, ENC1, ERCC3, EYA2, METTL21C, MIR584-6, NCOR2, PCBD2, RBMS3, RP1, SFPQ, SMAD6, SOX5, ZMYND8, ZNF792* |
| Processes related to development of embryo, fetus and cells | 28 | *ATRNL1, CD24, CDH13, EBF2, ELMO2, EPHA10, EYA2, FAM83B, FSTL5, GATA6, LAMA1, MEGF10, MSX2, NOL7, NSA2, PHACTR1, PITX1, PLXNA4, RBBP7, RBMS3, REPS2, SH2B3, SOBP, SOX5, SYNPO2, TP53RK, VPS13B, ZMYM4* |
| Miscellaneous | 65 | *AMMECR1L, B4GALT1, C3H1orf109, CCDC168, CCDC173, CRYBB2, CRYBB3, CUX2, DAO, DHX36, KIAA1671, KLHL23, LOC100137843, LOC100336909, LOC100847115, LOC100847998,* *LOC100848596,* *LOC101902700, LOC101904044, LOC101904842, LOC101905203, LOC101906469, LOC104969972, LOC104969973, LOC104970061, LOC104970733, LOC104973934, LOC104975198, LOC104975214, LOC107131937, LOC107133302, LOC112441475, LOC112442007, LOC112444209, LOC112444211, LOC112444897, LOC112445027, LOC112445112, LOC112445942, LOC112447073, LOC112447345, LOC112447641, LOC112447833, LOC112448046, LOC112449132, LOC112449242, LOC505052, LOC513508, LOC514680, LOC528043, LOC616281, LOC618220, LOC785669, LOC786055, LOC786974, NOL4, OBP, OPRM1, PHETA1, SGCD, SH3BGRL, SLC25A13, SLC35C2, SSBP2, TXNDC15* |

^1^Description of the shared function(s) of positional candidate genes for each group. ^2^ Number of genes in each group. ^3^List of positional candidate genes in each group.

LOC100848596

**Supplemental Table S3:** Relative risk for the most significant SNPs for the 86 loci associated with spontaneous abortion for primiparous cows bred by artificial insemination

| BTA^1^ | Position (Mb)^2^ | Locus #^3^ | Relative Risk^4^ |
| --- | --- | --- | --- |
| 1 | 113 | 1 | 1.73 |
| 1 | 120 | 2 | 3.37 |
| 1 | 128 | 3 | 4.12 |
| 1 | 128 | 4 | 2.42 |
| 1 | 129 | 5 | 2.07 |
| 2 | 5.1 | 6 | 2.57 |
| 2 | 26 | 7 | 3.62 |
| 2 | 60 | 8 | 2.67 |
| 2 | 66 | 9 | 2.53 |
| 3 | 14 | 10 | 1.34 |
| 3 | 108 | 11 | 3.95 |
| 3 | 110 | 12 | 1.48 |
| 4 | 13 | 13 | 3.53 |
| 4 | 17 | 14 | 1.07 |
| 4 | 95 | 15 | 3.73 |
| 5 | 85 | 16 | 1.81 |
| 6 | 6 | 17 | 3.30 |
| 6 | 61 | 18 | 1.58 |
| 7 | 6 | 19 | 3.53 |
| 7 | 14 | 20 | 1.48 |
| 7 | 46 | 21 | 1.25 |
| 7 | 68 | 22 | 2.73 |
| 7 | 81 | 23 | 3.94 |
| 7 | 89 | 24 | 1.36 |
| 7 | 92 | 25 | 1.68 |
| 8 | 74 | 26 | 1.39 |
| 8 | 87 | 27 | 3.73 |
| 8 | 104 | 28 | 1.68 |
| 8 | 107 | 29 | 1.78 |
| 9 | 28 | 30 | 1.03 |
| 9 | 42 | 31 | 1.25 |
| 9 | 47 | 32 | 1.83 |
| 9 | 90 | 33 | 1.74 |
| 10 | 13 | 34 | 1.79 |
| 10 | 95 | 35 | 1.56 |
| 10 | 98 | 36 | 1.32 |
| 12 | 52 | 37 | 4.12 |
| 12 | 78 | 38 | 1.48 |
| 13 | 6.1 | 39 | 1.88 |
| 13 | 75 | 40 | 2.06 |
| 13 | 75 | 41 | 3.94 |
| 14 | 22 | 42 | 1.12 |
| 14 | 55 | 43 | 2.80 |
| 14 | 65 | 44 | 2.70 |
| 14 | 67 | 45 | 1.60 |
| 16 | 16 | 46 | 2.02 |
| 16 | 53 | 64 | 1.76 |
| 16 | 36 | 47 | 1.54 |
| 17 | 36 | 48 | 3.75 |
| 17 | 51 | 49 | 3.11 |
| 17 | 55 | 50 | 3.58 |
| 17 | 60 | 51 | 1.97 |
| 17 | 64 | 52 | 1.25 |
| 17 | 65 | 53 | 2.02 |
| 17 | 65 | 54 | 1.60 |
| 17 | 9.1 | 55 | 2.37 |
| 18 | 18 | 56 | 1.48 |
| 18 | 23 | 57 | 1.97 |
| 18 | 33 | 58 | 1.93 |
| 18 | 41 | 59 | 1.67 |
| 18 | 45 | 60 | 1.18 |
| 18 | 7 | 61 | 1.84 |
| 20 | 11 | 62 | 2.21 |
| 20 | 16 | 63 | 2.14 |
| 20 | 1.9 | 64 | 1.97 |
| 22 | 3.9 | 65 | 1.43 |
| 22 | 5.5 | 66 | 1.44 |
| 23 | 43 | 67 | 1.34 |
| 23 | 23 | 68 | 6.99 |
| 24 | 34 | 69 | 3.43 |
| 24 | 35 | 70 | 1.72 |
| 24 | 35 | 71 | 1.81 |
| 24 | 35 | 72 | 1.56 |
| 24 | 37 | 73 | 2.41 |
| 24 | 40 | 74 | 1.47 |
| 24 | 14 | 75 | 1.42 |
| 25 | 17 | 76 | 3.75 |
| 25 | 31 | 77 | 1.69 |
| 26 | 36 | 78 | 1.68 |
| 26 | 7.6 | 79 | 1.93 |
| 28 | 9.5 | 80 | 1.42 |
| 28 | 39 | 81 | 1.52 |
| 28 | 48 | 82 | 1.08 |
| 29 | 71 | 83 | 2.57 |
| X | 127 | 84 | 1.60 |
| X | 137 | 85 | 1.72 |
| X | 113 | 86 | 1.73 |

^1^*Bos taurus* chromosome. In parentheses is the locus associated with spontaneous abortion that is numbered sequentially from chromosome 1 to the X chromosome so that the SNPs that comprise the loci associated with spontaneous abortion may be identified. ^2^ Location of SNP, in Megabases (Mb), measured by the numbered nucleotides in the ARS-UCD 1.2 reference genome assembly (https://www.ncbi.nlm.nih.gov/datasets/genome/GCF_002263795.1/). ^3^ The locus associated with spontaneous abortion that is numbered sequentially from chromosome 1 to the X chromosome so that the SNPs that comprise the loci associated with spontaneous abortion may be identified. ^4^ The calculated relative risk for the most significant SNP representing a locus.

**
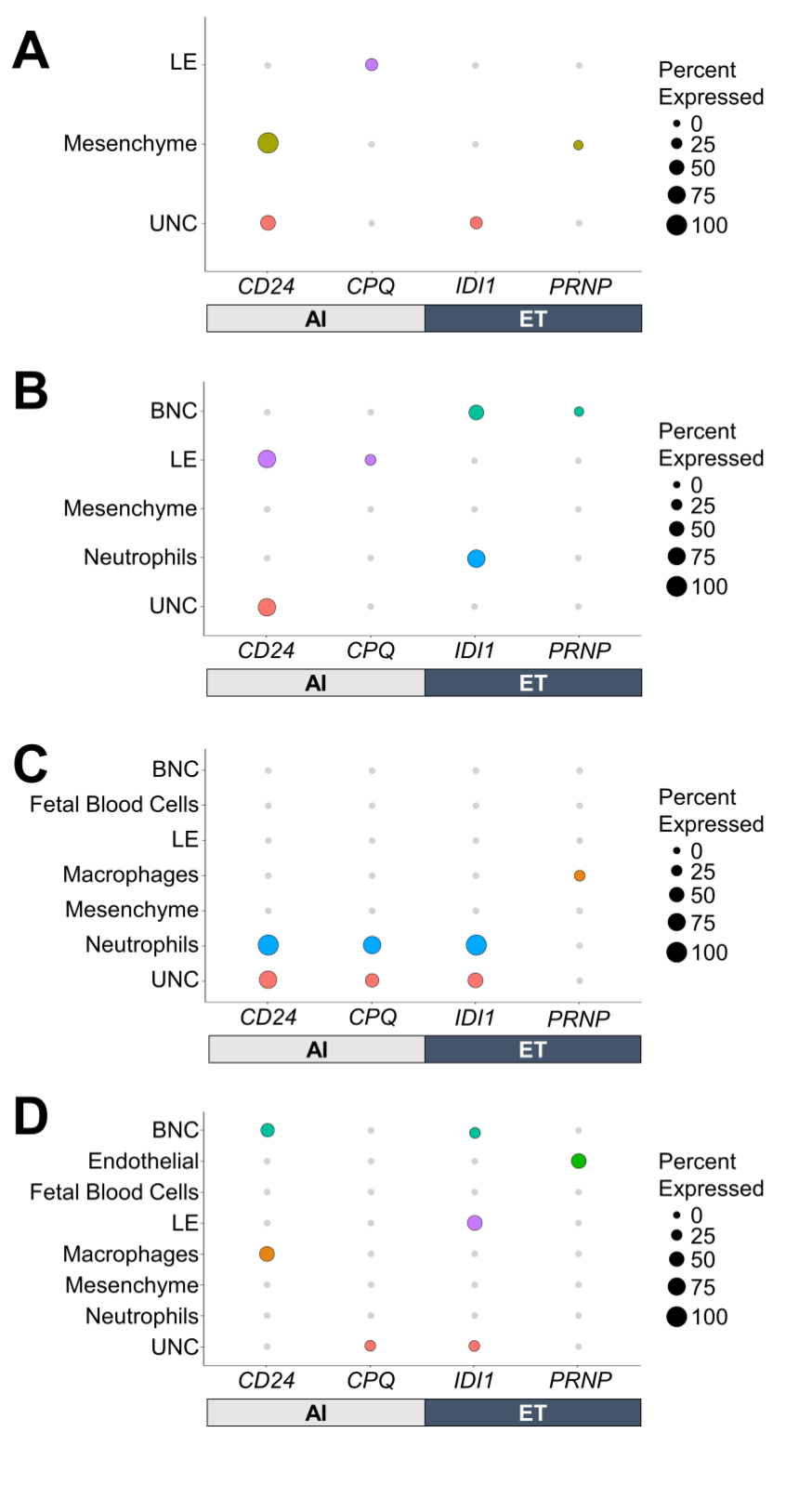
**

**Figure S2**: Expression of positional candidate genes associated with spontaneous abortion and expressed in multiple cattle single cell types at 17 (panel A), 24 (panel B), 30 (panel C), and 50 (panel D) days of gestation (Davenport et al., 2024). The cell types represented are binucleate cells (BNC), endothelial cells, fetal blood cells, endometrial epithelial cells (LE), macrophages, mesenchyme, neutrophils, and uninucleate cells (UNC).
